# Ambient Air Pollution and Parkinson’s Disease and Alzheimer’s Disease: An Updated Meta-Analysis

**DOI:** 10.3390/toxics13020139

**Published:** 2025-02-15

**Authors:** Cuiyao Xie, Xi Xia, Kai Wang, Jie Yan, Lijun Bai, Liqiong Guo, Xiaoxue Li, Shaowei Wu

**Affiliations:** 1 Department of Occupational and Environmental Health, School of Public Health, Xi’an Jiaotong University Health Science Center, Xi’an 710061, China; xiecuiyao@stu.xjtu.edu.cn (C.X.); xiaxi@xjtu.edu.cn (X.X.); wk10716@163.com (K.W.); jieyan509@163.com (J.Y.); bonheur-bai@stu.xjtu.edu.cn (L.B.); 2 Key Laboratory of Environment and Genes Related to Diseases, Ministry of Education, Xi’an Jiaotong University, Xi’an 710061, China; 3 Key Laboratory of Trace Elements and Endemic Diseases, Ministry of Health, Xi’an Jiaotong University, Xi’an 710061, China; 4 Key Laboratory for Disease Prevention and Control and Health Promotion of Shaanxi Province, Xi’an Jiaotong University, Xi’an 710061, China; 5 School of Disaster and Emergency Medicine, Tianjin University, Tianjin 300072, China; yingqidao@163.com; 6 Wenzhou Safety (Emergency) Institute, Tianjin University, Wenzhou 325000, China; 7 Disaster Medicine Research Center, Medical Innovation Research Division of the Chinese PLA General Hospital, Beijing 100853, China; 8 2021RU006 Research Unit of Disaster Medicine, Chinese Academy of Medical Sciences, Beijing 100005, China; 9 Beijing Key Laboratory of Disaster Medicine, Beijing 100039, China

**Keywords:** Alzheimer’s disease, ambient air pollution, meta-analysis, Parkinson’s disease, particulate matter

## Abstract

Background: Previous epidemiological evidence regarding the associations between ambient air pollution and two major neurodegenerative diseases, Alzheimer’s disease (AD) and Parkinson’s disease (PD), remains inconclusive. Objective: This study aimed to evaluate the associations between long-term and short-term exposure to PM_2.5_ and PM_10_ (i.e., particulate matter with an aerodynamic diameter of, or smaller than, 2.5 μm or 10 μm), nitrogen dioxide (NO_2_), ozone, sulfur dioxide, and carbon monoxide and the risks of AD and PD. Methods: A random-effects model was used to summarize individual effect estimates in the meta-analysis. A subgroup meta-analysis was further conducted to explore the potential sources of heterogeneity. Results: In total, 42 eligible studies were included. For each 5 μg/m^3^ increase in long-term PM_2.5_ exposure, the odds ratios (ORs) were 1.16 (95% CI: 1.04, 1.30; *I*^2^ = 95%) and 1.10 (95% CI: 1.03, 1.17; *I*^2^ = 95%) for AD and PD, respectively. For each 5 μg/m^3^ increase in short-term PM_2.5_ exposure, the OR was 1.01 (95% CI: 1.002, 1.01; *I*^2^ = 77%) for PD. For each 1 ppb increase in long-term NO_2_ exposure, the OR was 1.01 (95% CI: 1.0002, 1.02; *I*^2^ = 79%) for PD. Conclusion: Ambient air pollution, particularly PM_2.5_, may contribute to the increased risks of neurodegenerative diseases including AD and PD.

## 1. Introduction

Neurodegenerative diseases refer to a broad category of diseases that impact the neurons in the human brain. Neurodegenerative diseases occur when neurons are gradually lost, and they affect millions of people globally [1]. Among the 10 million neurodegenerative disease cases newly reported annually worldwide, the most prevalent form of dementia is Alzheimer’s disease (AD) [2,3,4]. Parkinson’s disease (PD) ranks second after AD [5]. For PD, the principal symptoms of motor include tremors, postural instability, and rigidity. The pathological features of PD include misfolded α-synuclein aggregation and dopaminergic neuron loss in the substantia nigra’s pars compacta [6]. Many factors influence the development of AD and PD. For the non-genetic hazard factors, advancing age consistently plays an important role in neurodegenerative diseases [7,8,9]. Furthermore, previous research indicated that the incidence rate of AD and PD may vary across gender and ethnicity/race [7,8,10,11,12]. The findings from a meta-analysis underscore the prospective effect of certain environmental factors on PD risk, comprising pesticide exposure, head trauma, and the consumption of well water [10,13,14].

Allied with cardiovascular and respiratory diseases, air pollution is an environmentally significant problem in modern societies [15,16,17]. However, the potential threat of air pollutants on neurodegenerative diseases has been overlooked until recently, despite increasing evidence indicating its detrimental effects on the brain [18,19]. Fine particulate matter (PM_2.5_) can trigger neuroinflammation by disrupting the blood–brain barrier [20,21]. Previous studies registered that exposure to elevated PM_2.5_ and ozone (O_3_), especially among young people and children, is linked to the high risk of AD development in the Metropolitan area of Mexico City [22,23].

Ambient air pollution poses a significant burden on public health. However, the effectiveness of protective measures often requires time to be implemented. Additionally, while current treatments for neurodegenerative diseases focus primarily on managing symptoms [24,25], most clinically used drugs only demonstrate effectiveness in the early disease stages. Effective drugs that target the underlying neuronal aspects to reverse AD or PD are currently lacking [26]. Therefore, it is crucial to provide an update in a timely manner in order to identify relevant hazardous air pollutants to prevent the occurrence of neurodegenerative diseases. In this updated meta-analysis, exposure to major ambient air pollutants is examined in relation to the risk of two major neurodegenerative diseases (AD and PD).

## 2. Methods

### 2.1. Databases and Search Strategies

This review was performed according to the recently published PRISMA guidelines (Table S1) [27]. We conducted a comprehensive search of relevant studies in Web of Science, PubMed, Scopus, and Embase between database inception and 5 March 2024 to assess the potential effects of ambient air pollutants on common neurodegenerative diseases (AD and PD). A wide range of search terms were used. The final searching strategy is enumerated in Table S2.

### 2.2. Study Selection

We imported all references from the databases into EndNote version 19.0.0.12062 for management. The duplicates were deleted using the software’s tool. Subsequently, we excluded the remaining articles that did not discuss the potential impacts of air pollution on AD or PD in our screening process. The remaining qualified articles were assessed using the same inclusion criteria. The screening and selection of studies were independently conducted by Cuiyao Xie and Jie Yan. Disagreements were resolved by a third investigator, Kai Wang, who provided the definitive verdict.

The criteria for inclusion were as follows: (1) epidemiological studies investigating the associations between the targeted ambient air pollutants with AD or PD; (2) studies providing quantitative estimates of risk measures including odd ratio (OR), hazard ratio (HR), and relative risk (RR) for the targeted ambient air pollutants in relation to AD or PD, along with their 95% confidence intervals (CIs) or sufficient data to calculate the estimates; and (3) studies published in English.

The criteria for exclusion were as follows: (1) studies categorized as reviews, editorials, case reports, conference abstracts, commentaries, or duplicate publications; (2) non-epidemiological studies such as in vitro or in vivo animal research, or botanical or toxicological studies; and (3) studies for which the specific estimates remained inaccessible, even after reaching out to the corresponding authors.

### 2.3. Data Extraction

Among all the articles mentioned above that met the inclusion and exclusion criteria, the extracted information was as follows: author, publication year, study age, area, period, cases, study design, air pollutant exposure assessment methods, estimated effect sizes, and 95% CIs. In cases where the quantitative data were absent from the articles, we contacted the corresponding authors for further clarification.

### 2.4. Quality Assessment

We used the Effective Public Health Practice Project (EPHPP, 2010) method to evaluate the rigor of epidemiological investigations with diverse designs using six key parameters in order to determine the quality of each eligible study. Two investigators (Cuiyao Xie and Jie Yan) conducted the quality assessment independently. A comprehensive description of the EPHPP is provided in Table S3.

### 2.5. Statistical Analyses

In epidemiological studies, HR is acknowledged as equivalent to RR with time-to-event analysis [28]. The value of OR to estimate RR has been considered suitable for diseases with low prevalence [29]. Given that both PD and AD have global prevalence rates under 5% [26,30,31,32,33], OR was the predominant measure of the exposure–health associations in our reviewed studies. We treated OR as equivalent to HR and RR for these conditions. The ORs from the included studies, which varied in exposure increment scales of ambient air pollutants, were standardized using the following formula: ORy = exp (ln (ORx) × (y/x)), where ‘x’ represents the pollutant exposure increase for the reported ORx in articles and ‘y’ represents the standardized increment in pollutant exposure for the calculated output ORy. We then computed the pooled risk estimates using a random-effects model. R statistical software was used for the statistical analyses (V 4.2.3).

For significant results pooled from more than ten studies, subgroup analyses were conducted to compare the pooled estimates by study area, female proportion, study design, age class, sample size, study quality, and exposure assessment.

The funnel plot is a straightforward graphical analysis tool designed to check for the existence of publication bias. However, funnel plots may not provide reliable insights when there are fewer than 10 studies. In this meta-analysis, many of the associations were found in less than 10 articles; therefore, the publication bias was determined using Egger’s and Begg’s tests. Furthermore, sensitivity analyses were performed by systematically omitting one study at a time.

## 3. Results

### 3.1. Results of the Search and Features of the Listed Studies

**Figure 1:**
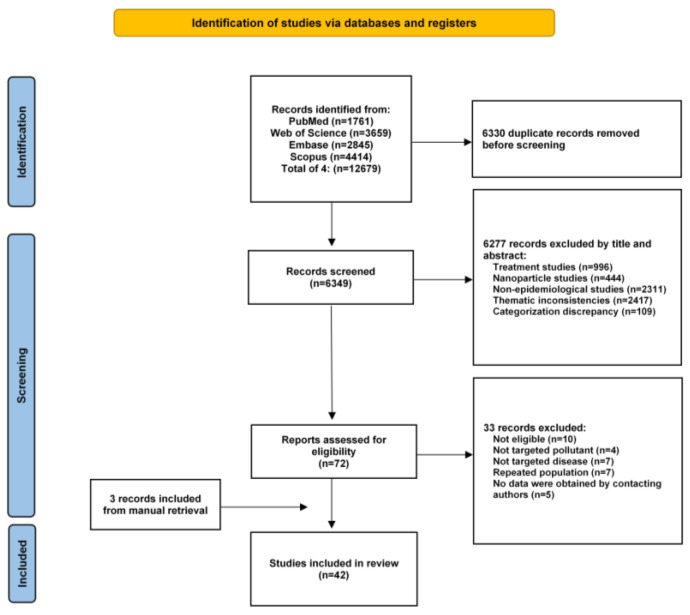
Flow diagram of the meta-analysis.

Among the 12,679 initially identified articles, 6349 titles and abstracts were reviewed after duplicate publications were removed. Of these, 6277 articles on irrelevant topics, including treatment studies, nanoparticle studies, non-epidemiological experiments, thematic inconsistencies, and categorization discrepancy (books, conferences, and non-English articles), were excluded. A total of 72 and 3 articles identified by manual search in previously published meta-analyses were reviewed carefully, and 42 eligible articles were finally included. The flow diagram illustrating our publication selection procedure is provided in Figure 1. The results of the quality assessment of these selected studies are presented in Tables S4 and S5.

Of these included articles, 23 were related to AD [34,35,36,37,38,39,40,41,42,43,44,45,46,47,48,49,50,51,52,53,54,55,56] and 25 were related to PD [38,40,42,46,52,53,57,58,59,60,61,62,63,64,65,66,67,68,69,70,71,72,73,74,75]. The predominant research type was cohort studies. A study [62] reported separate results for two states (North Carolina/Iowa), both of which were included simultaneously. The majority of studies focused on long-term exposure (Table S6).

As shown in Table 1 and Table 2, the publication years of the selected studies spanned the period from 2007 to 2024, encompassing research conducted across four geographical regions: Europe, Asia, North America, and Oceania.

### 3.2. Estimated Effects of Particulate Matter

#### 3.2.1. PM_2.5_

All results of the main meta-analyses are presented in Table 3, and all related forest plots are shown in the Supplementary Materials (Figures S1–S12). Our meta-analysis incorporated data from 21 studies for the association of PM_2.5_ with AD. Among these, 17 and 4 studies examined the associations between exposure (long-term and short-term) to PM_2.5_ and AD. The forest plot demonstrated that the pooled OR for the estimated effect of long-term PM_2.5_ (per 5 μg/m^3^ increase) exposure on AD was 1.16 (95% CI: 1.04, 1.30) (Figure S1), and the pooled OR for the estimated effect of short-term PM_2.5_ (per 5 μg/m^3^ increase) exposure on AD did not reach statistical significance [OR: 1.02 (95% CI: 0.99, 1.05)] (Figure S2).

**Table 1:** Characteristics of studies included in the meta-analysis for the association between ambient air pollution and risk of AD.

No.	Reference	Study Design/ Location	Study Period	Exposure Metric	Sample/ Cases	Age, Years	Pollutants	Exposure Assessment	Outcome Measure	Outcome Assessment and Instruments Used for Case
1	Carey et al. [34] (2018)	Cohort London	2005–2013	Long-term	130,978/ 848	50–79	PM_2.5_ NO_2_ O_3_	Model estimation	First recorded diagnosis	Medical data record (ICD-10) and physician-diagnosed
2	Cerza et al. [35] (2019)	Cohort Roma	2001–2013	Long-term	350,844/ 7669	74.5	PM_2.5_ PM_10_ NO_2_ O_3_	Model estimation	First hospitalization	Hospital records (ICD-9: 331.0)
3	Culqui et al. [36] (2017)	Time-series Madrid	2001–2009	Short-term (Lag2)	754,005	>60	PM_2.5_	Fixed monitoring site	Emergency hospital admission	ICD-9: 331.0
4	de Crom et al. [37] (2023)	Cohort Netherlands	2010–2018	Long-term	7511/ 406	>45	PM_2.5_ PM_10_ NO_2_	Model estimation	Newly diagnosed cases	MMSE, medical data record, and physician-diagnosed
5	Gandini et al. [38] (2018)	Cohort Italy	2001–2008	Long-term	74,989/ 248	>35	PM_2.5_ NO_2_	Model estimation	First hospitalization	Medical data record (ICD-9: 331)
6	Jung et al. [39] (2015)	Cohort Taiwan	2001–2010	Long-term	95,690/ 1399	>65	PM_2.5_ O_3_	Fixed monitoring site	Newly diagnosed cases	Medical data record (ICD-9: 331.0)
7	Kioumourtzoglou et al. [40] (2016)	Cohort America	1999–2010	Long-term	9,817,806/ 266,725	75.6	PM_2.5_	Fixed monitoring site	First hospital admission	Medical data record (ICD-9: 331.0)
8	Mortamais et al. [41] (2021)	Cohort French	1990–2012	Long-term	7066/ 541	73.4	PM_2.5_ NO_2_	Model estimation	Newly diagnosed cases	Physician-diagnosed (Diagnostic and Statistical Manual of Mental Disorders)
9	Nunez et al. [42] (2021)	Time-series America	2000–2014	Long-term	264,075	-	PM_2.5_	Model estimation	First hospitalization	Hospitalization records (ICD-9: 331.0)
10	Ran et al. [43] (2020)	Cohort Hongkong	1998–2011	Long-term	59,349/ 655	>65	PM_2.5_	Model estimation	First hospitalization	Hospitalization records (ICD-9: 290.0, 290.2, 290.3, 331.0)
11	Shaffer et al. [44] (2021)	Cohort Seattle	1994–2018	Long-term	4166/ 921	>65	PM_2.5_	Model estimation	Newly diagnosed cases	Physician-diagnosed
12	Shi et al. [46] (2020)	Cohort America	2000–2016	Long-term	63,038,019/2,490,431	>65	PM_2.5_	Model estimation	First hospitalization	Hospital data (ICD-9: 331.0; ICD-10: G30.9)
13	Shi et al. [45] (2021)	Cohort America	2000–2018	Long-term	12,456,447/804,668	>65	NO_2_ O_3_	Model estimation	Newly diagnosed cases	Medicare claims (ICD-9: 331.0; G30.0, G30.1, G30.8, G30.9)
14	Shim et al. [47] (2023)	Cohort Korea	2008–2019	Long-term	1,436,361	70.9	PM_10_	Fixed monitoring site	Newly diagnosed cases	ICD-10: F00, G30
15	Trevenen et al. [48] (2022)	Cohort Australia	1996–2018	Long-term	11,243/ 1670	72.1	PM_2.5_ NO_2_	Model estimation	Newly diagnosed cases	Self-reported, physician-diagnosed (ICD-9: 331.0, ICD-10: F00, G30)
16	Yang et al. [49] (2022)	Cohort China	2018–2020	Long-term	1545	68.21	PM_2.5_	Model estimation	Prevalence	Physician-diagnosed
17	Yang et al. [50] (2024)	Time-series China	2017–2019	Short-term (Lag1)	4975	79.82	PM_2.5_ PM_10_	Fixed monitoring site	Hospital admission	ICD-10: G30
18	Younan et al. [51] (2022)	Cohort America	1996–2010	Long-term	5798/ 130	>65	PM_2.5_	Model estimation	Newly diagnosed cases	Physician-diagnosed
19	Yuchi et al. [52] (2020)	Case–control Canada	1999–2003	Long-term	13,498/ 1227	45–84	PM_2.5_ NO_2_	Model estimation	Newly diagnosed cases	Hospital data (ICD-9: 331; ICD-10: G30)
20	Zanobetti et al. [53] (2014)	Case–crossover America	1999–2010	Short-term (lag02)	146,172	>65	PM_2.5_	Model estimation	Hospital admission	ICD-9: 331.0
21	Zhang et al. [55] (2022)	Cohort Britain	2006–2021	Long-term	227,840/ 1238	60.1	PM_2.5_ PM_10_ NO_2_	Model estimation	Newly diagnosed cases	Medical data record (ICD-9: 331.0; ICD-10: F00, F00.0, F00.1, F00.2, F00.9, G30, G30.0, G30.1, G30.8, G30.9)
22	Zhang et al. [54] (2023)	Case–crossover America	2005–2015	Short-term (lag03)	1,595,783	>45	PM_2.5_ NO_2_ O_3_	Model estimation	Emergency department visits	Hospital data
23	Zhu et al. [56] (2023)	Cohort China	2015–2022	Long-term	29,025/ 182	63.32	PM_2.5_ PM_10_ NO_2_	Model estimation	Newly diagnosed cases	Medical data record (ICD-10: G30)

Abbreviations: AD, Alzheimer’s disease; ICD, International Classification of Diseases; MMSE, Mini-Mental State Examination; NO_2_, nitrogen dioxide; O_3_, ozone; PM_2.5_, particulate matter with an aerodynamic diameter of 2.5 μm or smaller; PM_10_, particulate matter with an aerodynamic diameter of 10 μm or smaller.

**Table 2:** Characteristics of studies included in the meta-analysis for the association between ambient air pollution and risk of PD.

No.	Reference	Study Design/ Location	Study Period	Exposure Metric	Sample/ Cases	Age, Years	Pollutants	Exposure Assessment	Outcome Measure	Outcome Assessment and Instruments Used for Case
1	Cerza et al. [57] (2018)	Cohort Roma	2008–2013	Long-term	1,008,253/ 13,104	63	PM_2.5_ PM_10_ NO_2_ O_3_	Model estimation	Newly diagnosed cases	Administrative data (ICD-9: 332.0) and prescription
2	Chen et al. [58] (2017)	Case–control Taiwan	2000–2013	Long-term	54,524/ 1060	40–80	PM_10_ NO_2_ SO_2_ CO O_3_	Fixed monitoring site	Newly diagnosed cases	Physicians’ diagnoses (ICD-9: 332)
3	Finkelstein, Jerrett [59] (2007)	Case–control Hamilton/Toronto	1992–1999	Long-term	111,348/ 509	Birth year from 1900 to 1978	NO_2_	Model estimation	Newly diagnosed cases	Physicians’ diagnoses (ICD9: 332) and prescriptions for L-Dopa containing medications
4	Gandini et al. [38] (2018)	Cohort Itlay	2001–2008	Long-term	74,989/ 149	>35	PM_2.5_ NO_2_	Model estimation	First hospitalization	Medical data record (ICD-9: 332)
5	Goria et al. [60] (2021)	Time series France	2009–2017	Short-term (lag01)	196,479	-	PM_2.5_ PM_10_ NO_2_ O_3_	Fixed monitoring site	Hospital admission	ICD-10: G20, F023
6	Gu et al. [61] (2020)	Time series China	2013–2017	Short-term (lag1)	4,433,661	-	PM_2.5_ O_3_	Fixed monitoring site	Hospital admission	ICD-10
7	Kioumourtzoglou et al. [40] (2016)	Cohort America	1999–2010	Long-term	9,817,806/ 119,425	75.6	PM_2.5_	Fixed monitoring site	First hospital admission	Medical data record (ICD-9: 332)
8	Kirrane et al. [62] (2015)	Case–control North Carolina/Iowa	1993–2010	Long-term	83,343/ 301	12–92/42.77	PM_2.5_ O_3_	Model estimation	Newly diagnosed cases	Self-reported and physician-diagnosed
9	Lee et al. [65] (2016)	Case–control Taiwan	2007–2010	Long-term	55,585/ 11,117	72	PM_10_ SO_2_ CO O_3_	Model estimation	Newly diagnosed cases	Physicians’ diagnoses (ICD9: 332.0)
10	Lee et al. [64] (2017)	Case–crossover Korea	2002–2013	Short-term (lag07)	314	-	PM_2.5_ NO_2_ SO_2_ CO O_3_	Fixed monitoring site	Emergency admission case	Physician-diagnosed
11	Lee et al. [63] (2022)	Cohort Korea	2007–2015	Long-term	313,355/ 2621	48.9	PM_2.5_ PM_10_	Model estimation	Newly diagnosed cases	Hospital visit data (ICD-10: G20) and prescriptions
12	Liu et al. [66] (2016)	Case–control America	1995–2006	Long-term	4869	63.7	PM_2.5_ PM_10_ NO_2_	Model estimation	Newly diagnosed cases	Self-reported and physician-diagnosed
13	Nunez et al. [42] (2021)	Time series America	2000–2014	Long-term	114,514	-	PM_2.5_	Model estimation	First hospitalization	Hospitalization records (ICD-9: 332.0)
14	Palacios et al. [68] (2014)	Cohort America	1990–2008	Long-term	115,767/ 508	30–55	PM_2.5_ PM_10_	Model estimation	Newly diagnosed cases	Self-reported and physician-diagnosed
15	Palacios et al. [67] (2017)	Cohort America	1988–2010	Long-term	50,352/ 550	40–75	PM_2.5_ PM_10_	Model estimation	Newly diagnosed cases	Self-reported and physician-diagnosed
16	Ritz et al. [69] (2016)	Case–control Denmark	1996–2009	Long-term	3496/ 1696	62	NO_2_ CO	Model estimation	Newly diagnosed cases	Physician-diagnosed
17	Rumrich et al. [70] (2023)	Case–control Finland	1996–2015	Long-term	139,525/ 21,187	70.6	PM_2.5_ PM_10_	Model estimation	Newly diagnosed cases	ICD-10
18	Salimi et al. [71] (2019)	Cross-sectional Australia	2006–2009	Long-term	236,390/ 1428	62.5	PM_2.5_ NO_2_	Model estimation	Prevalence	Physician-diagnosed
19	Shi et al. [46] (2020)	Cohort America	2000–2016	Long-term	63,038,019/ 1,033,669	69.9	PM_2.5_	Model estimation	First hospitalization	Hospital data (ICD-9: 332; ICD-10: G20, G21.11, G21.19, G21.8)
20	Shin et al. [72] (2018)	Cohort Canada	2001–2013	Long-term	2,194,519/ 38,745	67	PM_2.5_ NO_2_ O_3_	Model estimation	Newly diagnosed cases	Hospital record
21	Toro et al. [73] (2019)	Case–control Netherlands	2010–2012	Long-term	1290/ 436	69	PM_2.5_ PM_10_ NO_2_	Model estimation	Newly diagnosed cases	Physician-diagnosed
22	Wei et al. [74] (2019)	Case–crossover America	2000–2012	Short-term (lag01)	214	-	PM_2.5_	Model estimation	Hospital admission	ICD-9
23	Yu et al. [75] (2021)	Cohort Ningbo	2015–2018	Long-term	47,516/ 206	62.27	PM_2.5_ PM_10_ NO_2_	Model estimation	Newly diagnosed cases	Medical data record (ICD-10: G20, G21.11, G21.19, G21.8)
24	Yuchi et al. [52] (2020)	Cohort Canada	1999–2003	Long-term	634,432/ 4201	58.1	PM_2.5_ NO_2_	Model estimation	Newly diagnosed cases	Physician claims recorded (332) and prescriptions
25	Zanobetti et al. [53] (2014)	Case–crossover America	1999–2010	Short-term (lag02)	40,496	>65	PM_2.5_	Model estimation	Hospital admission	ICD-9: 332

Abbreviations: CO, carbon monoxide; ICD, International Classification of Diseases; NO_2_, nitrogen dioxide; O_3_, ozone; PD, Parkinson’s disease; PM_2.5_, particulate matter with an aerodynamic diameter of 2.5 μm or smaller; PM_10_, particulate matter with an aerodynamic diameter of 10 μm or smaller; SO_2_, sulfur dioxide.

**Table 3:** The results of the meta-analysis for the association between ambient air pollution and risk of AD or PD.

Outcome	Pollutant	Exposure Duration	No. of Estimates	OR	95%CI	*p*-Value
**AD**	PM_2.5_	Long-term	17	1.16	1.04, 1.30	0.010
	PM_2.5_	Short-term	4	1.02	0.99, 1.05	0.185
	PM_10_	Long-term	5	1.03	0.96, 1.10	0.411
	NO_2_	Long-term	10	1.01	0.99, 1.02	0.455
	O_3_	Long-term	4	0.9998	0.99, 1.01	0.954
**PD**	PM_2.5_	Long-term	17	1.10	1.03, 1.17	0.003
	PM_2.5_	Short-term	5	1.01	1.002, 1.01	0.016
	PM_10_	Long-term	10	0.99	0.99, 1.004	0.235
	NO_2_	Long-term	11	1.01	1.0002, 1.02	0.045
	O_3_	Long-term	6	1.003	0.9998, 1.01	0.065
	O_3_	Short-term	3	1.002	0.999, 1.005	0.284
	CO	Long-term	3	1.32	0.82, 2.11	0.255

Abbreviations: AD, Alzheimer’s disease; CO, carbon monoxide; NO_2_, nitrogen dioxide; O_3_, ozone; PD, Parkinson’s disease; PM_2.5_, particulate matter with an aerodynamic diameter of 2.5 μm or smaller; PM_10_, particulate matter with an aerodynamic diameter of 10 μm or smaller.

For PD, our meta-analysis incorporated data from 21 studies. Among these, 16 and 5 studies reported the associations between long-term and short-term PM_2.5_ exposure and PD, respectively. The forest plots revealed that the pooled ORs for the estimated effects of long-term and short-term PM_2.5_ (per 5 μg/m^3^ increase) exposure on PD were 1.10 (95%CI: 1.03, 1.17) and 1.01 (95%CI: 1.002, 1.01) (marginal significance), respectively (Figures S3 and S4).

#### 3.2.2. Particulate Matter with a Diameter Smaller than 10 μm (PM_10_)

Six studies explored the association between PM_10_ and AD, with five and one study focusing on the potential effects of long-term and short-term exposure, respectively. Eleven studies investigated the association between PM_10_ and PD, with ten and one study focusing on the potential effects of long-term and short-term exposure, respectively. The pooled ORs for the estimated effects of long-term PM_10_ (per 5 μg/m^3^ increase) exposure on AD [OR: 1.03 (95% CI: 0.96, 1.10)] (Figure S5) and PD [OR: 0.99 (95% CI: 0.99, 1.004)] (Figure S6) did not reach statistical significance.

### 3.3. Estimated Effects of Gaseous Pollutants

#### 3.3.1. NO_2_

Eleven studies investigated the association between NO_2_ and AD. Among these, 10 and 1 study examined the associations, respectively, between long-term and short-term exposure to NO_2_ and AD. No significant association was found between long-term NO_2_ exposure and the risk of AD (Figure S7). Thirteen studies investigated the association between NO_2_ and PD. Among these, 11 and 2 studies reported the associations between long-term and short-term NO_2_ exposure and PD, respectively. The forest plot demonstrated that the pooled OR for the estimated effects of long-term NO_2_ (per 1 ppb increase) exposure on PD was 1.01 (95% CI: 1.0002, 1.02), with marginal significant association (Figure S8).

#### 3.3.2. O_3_

Five studies explored the association between O_3_ and AD, and four studies focused on the potential estimated effects of long-term exposure. Eight studies investigated the association between O_3_ and PD, with five and three studies focusing on the potential effects of long-term and short-term exposure, respectively. No significant association was found for O_3_. The forest plots demonstrated that the pooled ORs for the estimated effects of long-term O_3_ (per 1 ppb increase) exposure on AD was 0.9998 (95% CI: 0.99, 1.01), long-term O_3_ exposure on PD was 1.003 (95% CI: 0.9998, 1.01), and short-term O_3_ exposure on PD was 1.002 (95% CI: 0.999, 1.005) (Figures S9–S11, respectively).

#### 3.3.3. Sulfur Dioxide (SO_2_)

Only three studies investigated the association between SO_2_ and PD (two on long-term effects and one on short-term effects), and no studies investigated the association between SO_2_ and AD. The limited studies were insufficient for a meaningful meta-analysis.

#### 3.3.4. Carbon Monoxide (CO)

Only four studies investigated the association between CO and PD (three on long-term effects and one on short-term effects), and no studies investigated the association between CO and AD. However, the association for long-term CO exposure (per 1 mg/m^3^ increase) and PD did not reach statistical significance (Figure S12).

### 3.4. Subgroup Analysis

#### 3.4.1. PM_2.5_ Exposure and AD

Regarding the association between PM_2.5_ exposure and AD, we observed some notable subgroup heterogeneity. The heterogeneity among studies for long-term exposure to PM_2.5_ can be partly explained by female proportion (*p* = 0.008). While studies with less than 50% females did not show significant association between PM_2.5_ and the risk of AD [OR: 1.01, (95%CI: 0.98, 1.04)], studies with more than 50% females suggested that PM_2.5_ (per 5 μg/m^3^ increase) was associated with an increased risk of AD [OR: 1.22, (95% CI: 1.07,1.38)] (Table S7).

The heterogeneity among studies for short-term exposure to PM_2.5_ can be partly explained by study area (*p* < 0.001). Compared to studies in North America, studies in both Asia [OR: 1.02, (95% CI: 1.01, 1.03)] and Europe [OR: 1.08, (95% CI: 1.04, 1.13)] showed a more obvious association of PM_2.5_ (per 5 μg/m^3^ increase) with increased risks of AD (Table S7).

#### 3.4.2. PM_2.5_ Exposure and PD

For the association between long-term PM_2.5_ exposure and PD, we also observed heterogeneity between subgroups divided by study area (*p* < 0.001), female proportion (*p* = 0.008), study design (*p* < 0.001), and exposure assessment (*p* = 0.001). While studies in Europe and Oceania did not show a significant association of PM_2.5_ exposure with PD, studies in both Asia [OR: 1.17, (95% CI: 1.07, 1.29)] and North America [OR: 1.15, (95% CI: 1.04, 1.28)] indicated that the per 5 μg/m^3^ increase in PM_2.5_ was associated with an increased risk of PD. Additionally, studies with more than 50% females [OR: 1.11, (95% CI: 1.03, 1.20)], with cohort [OR: 1.10, (95% CI: 1.02, 1.18)] and time-series [OR: 1.54, (95% CI: 1.22, 1.94)] designs, and with exposure assessment of a fixed site [OR: 1.47, (95% CI: 1.22, 1.77)] showed an increased risk of PD associated with the per 5 μg/m^3^ increase in PM_2.5_ (Table S7).

The heterogeneity among studies for short-term PM_2.5_ exposure can be partly explained by study area (*p* = 0.038), sample size (*p* = 0.002), study design (*p* = 0.002), exposure assessment (*p* = 0.002), and study quality (*p* = 0.002). While studies in North America [OR: 1.11, (95% CI: 0.88, 1.39)] did not show a significant association of PM_2.5_ exposure with PD, studies in Asia [OR: 1.02, (95% CI: 1.01, 1.02)] and Europe [OR: 1.00, (95% CI: 1.00, 1.01)] indicated that the per 5 μg/m^3^ increase in PM_2.5_ was associated with an increased risk of PD, respectively. Additionally, studies with a sample size less than 100,000 [OR: 1.02, (95% CI: 1.01, 1.02)], with a case–control design [OR: 1.02, (95% CI: 1.01, 1.02)], with exposure assessment based on model prediction [OR: 1.02, (95% CI: 1.01, 1.02)], and with high study quality [OR: 1.02, (95% CI: 1.01, 1.02)] showed an increased risk of PD associated with the per 5 μg/m^3^ increase in PM_2.5_ (Table S7).

#### 3.4.3. The Association Between NO_2_ Exposure, and AD and PD

For the association of NO_2_ with AD, the heterogeneity among studies for long-term NO_2_ exposure can be partly explained by age group (*p* = 0.004), female proportion (*p* = 0.034), and study design (*p* = 0.025). Notably, while studies with a mean age of more than 65 years old showed no significant association between NO_2_ and the risk of AD [OR = 0.99, (95%CI: 0.98, 1.01)], studies with a mean age less than 65 years old showed that the per 1 ppb increase in NO_2_ was associated with an increased risk of AD [OR = 1.03, (95% CI: 1.01, 1.05)]. Regarding the association of NO_2_ with PD, no significant factors have been identified in the subgroup analyses (Table S8).

### 3.5. Publication Bias and Sensitivity Analyses

All funnel plots are available in the Supplementary Materials (Figures S13–S18). Egger’s and Begg’s tests, except for the association between long-term PM_10_ and NO_2_ exposure, and PD (Table S9), showed no noteworthy publication bias for the associations investigated. The exclusion of any specific study did not result in significant alterations in the original meta-analysis results across various air pollutants (Table S10).

## 4. Discussion

### 4.1. Summary of Results

Overall, in this comprehensive review of 42 studies across diverse regions, the findings demonstrated a significant association between long-term PM_2.5_ exposure and an increased risk of AD and PD, and that short-term exposure to PM_2.5_ was associated with a significantly increased risk of PD. These findings suggest that PM_2.5_ may be a potential risk factor for both AD and PD. However, our investigation did not reveal any significant association of PM_10_ with AD or PD. Additionally, long-term NO_2_ exposure was found to be associated with a marginally significant increase in the risk of PD. In contrast, our analysis did not find significant results for O_3_, SO_2_, and CO, likely due to the paucity of available studies, making it challenging to reach reliable conclusions.

Several recent meta-analyses have shed light on the link between air pollution and neurodegenerative diseases. Cheng et al. [76] conducted a search across four databases, spanning from January 1900 to June 2022, to investigate the associations between long-term PM_2.5_ exposure, and AD and vascular dementia (VaD). Twenty articles were included in their meta-analysis, of which thirteen and eight studies investigated associations of long-term PM_2.5_ exposure with AD and VaD, respectively, and the meta-analysis identified significant pooled effect estimates for long-term PM_2.5_ exposure, and both AD and VaD. Similarly, Gong et al. [77] investigated the association between long-term PM_2.5_ exposure and neurodegenerative diseases (including 17 studies for dementia, 13 for AD, 8 for PD, 8 for VaD, 2 for amyotrophic lateral sclerosis, and 14 for cognitive decline) and identified 4, 3, and 5 studies for the associations between long-term PM_10_ exposure with all-cause dementia, AD, and PD, respectively. They only found a pooled positive association between long-term PM_2.5_ (per 10 μg/m^3^ increase) exposure and AD [OR = 1.65, (95%CI: 1.37, 1.94)] from 13 studies and a marginally significant association with PD [OR = 1.17, (95%CI: 1.0, 1.33)] from eight studies. This investigation, similarly, did not reveal any significant association of PM_10_ with AD or PD. This may be due to the larger particle size of PM_10_, which may result in less nervous system damage compared to PM_2.5_. Future research is required to validate the potential effects of PM_10_ on these neurodegenerative diseases. It is worth noting that both the above meta-analyses exclusively focused on long-term exposure and overlooked the broader spectrum of air pollutants and the potential impact of short-term exposure. Furthermore, studies conducted in regions with high PM_2.5_ concentrations, particularly Asia, remain inadequate. It is crucial to gather more substantial data from Asian areas because the majority of PM_2.5_-associated premature deaths occurred in Asia [78]. However, the studies from China included in previous meta-analyses were only confined to Hong Kong [43,79] and Taiwan [39]. In the present study, we have incorporated studies from more inland provinces of China, such as Sichuan [50] and Ningbo [56,75].

Dhiman et al. [5] conducted a comprehensive meta-analysis of the associations between major ambient air pollutants and PD, and they identified 6, 4, 6, 4, 2, and 4 eligible studies for the association between PM_2.5_, PM_10_, NO_2,_ O_3_, SO_2_, and CO exposure (not differentiating long-term and short-term exposure) and PD, respectively. The results showed marginal significant associations between NO_2_ [OR = 1.01, (95% CI: 1.00, 1.02)] and O_3_ [OR = 1.01, (95% CI: 1.00, 1.02)], and PD. However, the above meta-analysis only covered studies published up to 2019; hence, it is necessary to update the meta-analysis due to the limited number of studies included in previous meta-analyses. In order to ascertain the potential effects of exposure to pollutants across different timeframes, we conducted meta-analyses for long-term and short-term exposure, respectively. Our analysis identified 16, 10, 11, 5, 2, and 3 eligible studies for the associations between long-term PM_2.5_, PM_10_, NO_2,_ O_3_, SO_2_, and CO exposure and PD and identified 5, 1, 2, 3, 1, and 1 eligible study for the associations between short-term PM_2.5_, PM_10_, NO_2,_ O_3_, SO_2_, and CO exposure and PD, respectively. We also newly identified a marginally significant association between long-term NO_2_ exposure and PD. In contrast, we did not find any significant association between long-term O_3_ exposure and PD with an increased number of included studies as well.

### 4.2. Biological Plausibility

Particulate matter (PM), known to induce various central nervous system pathologies, is a complex mixture. Increasing evidence suggests the prominent effects of PM include oxidative stress, neuroinflammation, endoplasmic reticulum stress, dysfunction of mitochondria, and disturbance of protein homeostasis [78,80]. Exposure to PM_2.5_ in rodents has been linked to significant increases in the expressions of oxidative stress-related genes or markers [81]. One possible explanation is that the existence of PM in the brain initially triggers microglia activation, which produces excessive reactive oxygen species [82,83]. Given that the brain is a high energy-consuming organ, it is particularly sensitive to oxidative stress. When a disturbance in the redox balance occurs, it could eventually erupt the blood–brain barrier [73]. Once PM enters the body, it also triggers the release of pro-inflammatory mediators, which can result in systemic inflammation and the development of chronic respiratory issues [84], which has detrimental effects on the nervous system [78,85]. This stimuli-produced reactive oxygen species production can reduce the expression of tight-junction protein, leading to blood–brain barrier damage and contributing to amyloid-β42 accumulation in the olfactory bulb and frontal cortex in exposed prepuberal children [86,87]. Additionally, stimulation of PM consumedly disturbs the homeostasis of intracellular organelles such as mitochondria and the endoplasmic reticulum. Excessive endoplasmic reticulum stress can initiate apoptosis [88]. Air pollutants may contribute to the development of PD through mechanisms such as mitochondrial dysfunction, which increases the vulnerability of dopaminergic neurons [89]. Concisely, all of these potentially indefinite mechanisms are not mutually exclusive but may be complementary and may interact with each other.

In addition to PM, elevated long-term exposure to NO_2_, SO_2_, and CO have also been linked with an increased risks of cognitive decline and AD-like pathological abnormalities [81,90,91,92,93]. It is possible that NO_2_ exerts direct toxic effects on the brain. A study has demonstrated that inhalation of NO_2_ (5 h/d, 4 weeks) is associated with an increase in the accumulation of amyloid-β42 and a decline in cognitive function [92]. In addition, a retrospective cohort study showed that long-term NO_2_ exposure was significantly associated with the onset of PD [94]. Particularly, increased oxidative stress and mitochondrial injury and dysfunction may be a noteworthy mechanism that contributes to the development of neurodegenerative disorders following exposure to NO_2_ [92,95].

### 4.3. Strengths and Limitations

This study has certain strengths. First, this study provides the most comprehensive assessment of the associations between exposure (both long-term and short-term) to ambient air pollution and major neurodegenerative diseases (both AD and PD). Second, we focused on not only particulate matters but also gaseous pollutants. Third, we provide the latest updated review that includes much more studies, especially from a high-polluted area (e.g., Asia).

However, some limitations of the included studies need to be taken into consideration. First, investigations into the relationship between exposure to ambient air pollution and targeted diseases are relatively limited, which makes it difficult to draw firm conclusions from this meta-analysis. Second, many available studies on the associations between air pollutants and AD or PD are observational studies, which may suggest associations between these air pollutants and the disease outcomes but do not precisely confirm causal associations. Third, it lacks data from highly polluted regions such as South America and Africa, where PM_2.5_ exposure is a significant public health concern. In order to enhance the representativeness of future studies and ensure the generalizability and applicability of their findings, it is important to conduct more cohort and intervention studies, especially in highly polluted regions. Fourth, this study does not incorporate diverse methodologies for precise exposure assessment, which may affect the accuracy of the results. To enhance the robustness of evidence in this field, there is an urgent need for future research to employ advanced exposure assessment techniques such as personal monitoring or satellite exposure assessments.

## 5. Conclusions

In conclusion, our meta-analysis identified that exposure to the major air pollutant PM_2.5_ was associated with increased risks of two major neurodegenerative diseases (i.e., AD and PD). Expanding studies in underrepresented regions and elucidating the molecular mechanisms underlying the neurotoxic effects of ambient air pollution will be crucial for governments to make relevant public health policies.

## Data Availability

The data that support the findings of this study are available from the corresponding authors upon reasonable request.

## Supplementary Materials

The following supporting information can be downloaded at https://www.mdpi.com/article/10.3390/toxics13020139/s1, Figure S1: Forest plot for the meta-analysis on the association between long-term exposure PM_2.5_ and risk of AD; Figure S2: Forest plot for the meta-analysis on the association between short-term exposure PM_2.5_ and risk of AD; Figure S3: Forest plot for the meta-analysis on the association between long-term exposure PM_2.5_ and risk of PD; Figure S4: Forest plot for the meta-analysis on the association between short-term exposure PM_2.5_ and risk of PD; Figure S5: Forest plot for the meta-analysis on the association between long-term exposure PM_10_ and risk of AD; Figure S6: Forest plot for the meta-analysis on the association between long-term exposure PM_10_ and risk of PD; Figure S7: Forest plot for the meta-analysis on the association between long-term exposure NO_2_ and risk of AD; Figure S8: Forest plot for the meta-analysis on the association between long-term exposure NO_2_ and risk of PD; Figure S9: Forest plot for the meta-analysis on the association between long-term exposure O_3_ and risk of AD; Figure S10: Forest plot for the meta-analysis on the association between long-term exposure O_3_ and risk of PD; Figure S11: Forest plot for the meta-analysis on the association between short-term exposure O_3_ and risk of PD; Figure S12: Forest plot for the meta-analysis on the association between long-term exposure CO and risk of PD; Figure S13: Funnel plot for the meta-analysis on the associations between long-term PM_2.5_ exposure (per 5 μg/m^3^ increase) and risk of AD; Figure S14: Funnel plot for the meta-analysis on the associations between long-term NO_2_ exposure (per 1ppb increase) and risk of AD; Figure S15: Funnel plot for the meta-analysis on the associations between long-term PM_2.5_ exposure (per 5 μg/m^3^ increase) and risk of PD; Figure S16: Funnel plot for the meta-analysis on the associations between long-term PM_10_ exposure (per 5 μg/m^3^ increase) and risk of PD; Figure S17: Funnel plot for the meta-analysis on the associations between long-term NO_2_ exposure (per 1ppb increase) and risk of PD; Figure S18: Funnel plot for the meta-analysis on the associations between long-term O_3_ exposure (per 1ppb increase) and risk of PD; Table S1: PRISMA 2020 checklist; Table S2: The detailed search strategy of the meta-analysis; Table S3: Explanatory file for Effective Public Health Practice Project (EPHPP) Quality Assessment Tool; Table S4: Quality assessment using the Effective Public Health Practice Project (EPHPP) Quality Assessment Tool for the included studies for AD; Table S5: Quality assessment using the Effective Public Health Practice Project (EPHPP) Quality Assessment Tool for the included studies for PD; Table S6: The detailed quantity of estimate included in the meta-analyses; Table S7: Subgroup analysis for the meta-analysis on the association between PM_2.5_ exposure and risk of AD or PD; Table S8: Subgroup analysis for the meta-analysis on the association between NO_2_ exposure and risk of AD or PD; Table S9: Estimated publication bias in the association between exposure to each pollutant and risk of PD or AD when the number of studies reached above five; Table S10: Sensitivity analyses for the meta-analysis.
