# Supplementary material for: Ambient Air Pollution and Parkinson’s Disease and Alzheimer’s Disease: An Updated Meta-Analysis
Source: Toxics. 2025 Feb 15;13(2):139. doi: 10.3390/toxics13020139 (PMC11861764; doi:10.3390/toxics13020139)
Supplement: Supplementary file 1 [file toxics-13-00139-s001.zip › toxics-3458556-supplementary.pdf]

Supplementary material
Ambient Air Pollution and Parkinson's Disease and Alzheimer's Disease: An Updated Meta-Analysis.

Cuiyao Xie, Xi Xia, Kai Wang, Jie Yan, Lijun Bai, Liqiong Guo, Xiaoxue Li, Shaowei Wu

Table of contents

Table S1. PRISMA 2020 checklist.
Table S2. The detailed search strategy of the meta-analysis.
Table S3. Explanatory file for Effective Public Health Practice Project (EPHPP) Quality Assessment Tool.
Table S4. Quality assessment using the Effective Public Health Practice Project (EPHPP) Quality Assessment Tool for the included studies for AD.
Table S5. Quality assessment using the Effective Public Health Practice Project (EPHPP) Quality Assessment Tool for the included studies for PD.
Table S6. The detailed quantity of estimate included in the meta-analyses.
Table S7. Subgroup analysis for the meta-analysis on the association between $PM_{2.5}$ exposure and risk of AD or PD.
Table S8. Subgroup analysis for the meta-analysis on the association between NO_2 exposure and risk of AD or PD.
Table S9. Estimated publication bias in the association between exposure to each pollutant and risk of PD or AD when the number of studies reached above five.
Table S10. Sensitivity analyses for the meta-analysis.
Figure S1. Forest plot for the meta-analysis on the association between long-term exposure $PM_{2.5}$ and risk of AD.
Figure S2. Forest plot for the meta-analysis on the association between short-term exposure $PM_{2.5}$ and risk of AD.
Figure S3. Forest plot for the meta-analysis on the association between long-term exposure $PM_{2.5}$ and risk of PD.
Figure S4. Forest plot for the meta-analysis on the association between short-term exposure $PM_{2.5}$ and risk of PD.
Figure S5. Forest plot for the meta-analysis on the association between long-term exposure PM_{10} and risk of AD.
Figure S6. Forest plot for the meta-analysis on the association between long-term exposure PM_{10} and risk of PD.
Figure S7. Forest plot for the meta-analysis on the association between long-term exposure NO_2 and risk of AD.
Figure S8. Forest plot for the meta-analysis on the association between long-term exposure NO_2 and risk of PD.
Figure S9. Forest plot for the meta-analysis on the association between long-term exposure O_3 and risk of AD.
Figure S10. Forest plot for the meta-analysis on the association between long-term exposure O_3

and risk of PD.

Figure S11. Forest plot for the meta-analysis on the association between short-term exposure O_3 and risk of PD.

Figure S12. Forest plot for the meta-analysis on the association between long-term exposure CO and risk of PD.

Figures S13-S18. Funnel plot for meta-analysis of studies analyzing the publication bias when the number of studies reached above five.

Table S1. PRISMA 2020 checklist.

Section and Topic	Item #	Checklist item	Location where item is reported
**TITLE**			
Title	1	Identify the report as a meta-analysis.	1
**ABSTRACT**			
Abstract	2	See the PRISMA 2020 for Abstracts checklist.	1
**INTRODUCTION**			
Rationale	3	Describe the rationale for the review in the context of existing knowledge.	1-2
Objectives	4	Provide an explicit statement of the objective(s) or question(s) the review addresses.	2
**METHODS**			
Eligibility criteria	5	Specify the inclusion and exclusion criteria for the review and how studies were grouped for the syntheses.	2-3
Information sources	6	Specify all databases, registers, websites, organisations, reference lists and other sources searched or consulted to identify studies. Specify the date when each source was last searched or consulted.	2
Search strategy	7	Present the full search strategies for all databases, registers and websites, including any filters and limits used.	Supplementary material Table S2
Selection process	8	Specify the methods used to decide whether a study met the inclusion criteria of the review, including how many reviewers screened each record and each report retrieved, whether they worked independently, and if applicable, details of automation tools used in the process.	3
Data collection process	9	Specify the methods used to collect data from reports, including how many reviewers collected data from each report, whether they worked independently, any processes for obtaining or confirming data from study investigators, and if applicable, details of automation tools used in the process.	2-3

Section and Topic	Item #	Checklist item	Location where item is reported
Data items	10a	List and define all outcomes for which data were sought. Specify whether all results that were compatible with each outcome domain in each study were sought (e.g. for all measures, time points, analyses), and if not, the methods used to decide which results to collect.	NA
	10b	List and define all other variables for which data were sought (e.g. participant and intervention characteristics, funding sources). Describe any assumptions made about any missing or unclear information.	NA
Study risk of bias assessment	11	Specify the methods used to assess risk of bias in the included studies, including details of the tool(s) used, how many reviewers assessed each study and whether they worked independently, and if applicable, details of automation tools used in the process.	2-3
Effect measures	12	Specify for each outcome the effect measure(s) (e.g. risk ratio, mean difference) used in the synthesis or presentation of results.	3
Synthesis methods	13a	Describe the processes used to decide which studies were eligible for each synthesis (e.g. tabulating the study intervention characteristics and comparing against the planned groups for each synthesis (item #5)).	2-4 & Figure 1
	13b	Describe any methods required to prepare the data for presentation or synthesis, such as handling of missing summary statistics, or data conversions.	3
	13c	Describe any methods used to tabulate or visually display results of individual studies and syntheses.	3
	13d	Describe any methods used to synthesize results and provide a rationale for the choice(s). If meta-analysis was performed, describe the model(s), method(s) to identify the presence and extent of statistical heterogeneity, and software package(s) used.	3
	13e	Describe any methods used to explore possible causes of heterogeneity among study results (e.g. subgroup analysis, meta-regression).	3

Section and Topic	Item #	Checklist item	Location where item is reported
	13f	Describe any sensitivity analyses conducted to assess robustness of the synthesized results.	3
Reporting bias assessment	14	Describe any methods used to assess risk of bias due to missing results in a synthesis (arising from reporting biases).	3
Certainty assessment	15	Describe any methods used to assess certainty (or confidence) in the body of evidence for an outcome.	3
**RESULTS**			
Study selection	16a	Describe the results of the search and selection process, from the number of records identified in the search to the number of studies included in the review, ideally using a flow diagram.	4
	16b	Cite studies that might appear to meet the inclusion criteria, but which were excluded, and explain why they were excluded.	4 & Figure 1
Study characteristics	17	Cite each included study and present its characteristics.	4 & Tables 1-2
Risk of bias in studies	18	Present assessments of risk of bias for each included study.	Supplementary material Tables S4-S5
Results of individual studies	19	For all outcomes, present, for each study: (a) summary statistics for each group (where appropriate) and (b) an effect estimate and its precision (e.g. confidence/credible interval), ideally using structured tables or plots.	4, 12 & Table 3
Results of syntheses	20a	For each synthesis, briefly summarise the characteristics and risk of bias among contributing studies.	4, 12-13
	20b	Present results of all statistical syntheses conducted. If meta-analysis was done, present for each the summary estimate and its precision (e.g. confidence/credible interval) and measures of statistical heterogeneity. If comparing groups, describe the direction of the effect.	4, 12 & Table 3

Section and Topic	Item #	Checklist item	Location where item is reported
	20c	Present results of all investigations of possible causes of heterogeneity among study results.	13
	20d	Present results of all sensitivity analyses conducted to assess the robustness of the synthesized results.	Supplementary material Table S10
Reporting biases	21	Present assessments of risk of bias due to missing results (arising from reporting biases) for each synthesis assessed.	14 & Supplementary material Table S9
Certainty of evidence	22	Present assessments of certainty (or confidence) in the body of evidence for each outcome assessed.	14 & Supplementary material Table S9
**DISCUSSION**			
Discussion	23a	Provide a general interpretation of the results in the context of other evidence.	15
	23b	Discuss any limitations of the evidence included in the review.	16
	23c	Discuss any limitations of the review processes used.	16
	23d	Discuss implications of the results for practice, policy, and future research.	16
**OTHER INFORMATION**			
Registration and protocol	24a	Provide registration information for the review, including register name and registration number, or state that the review was not registered.	NA
	24b	Indicate where the review protocol can be accessed, or state that a protocol was not prepared.	NA
	24c	Describe and explain any amendments to information provided at registration or in the protocol.	NA
Support	25	Describe sources of financial or non-financial support for the review, and the role of the funders or sponsors in the review.	17

Section and Topic	Item #	Checklist item	Location where item is reported
Competing interests	26	Declare any competing interests of review authors.	17
Availability of data, code and other materials	27	Report which of the following are publicly available and where they can be found: template data collection forms; data extracted from included studies; data used for all analyses; analytic code; any other materials used in the review.	17

Table S2. The detailed search strategy of the meta-analysis.

Database	Literature Search strategy
**1. PubMed**	#1 "Air Pollution"[Mesh] OR "Air Pollution" OR "Air pollutant*" OR "Air Quality" OR "Carbon monoxide" OR "Sulfur dioxide" OR "Nitrogen dioxide" OR "Ozone" OR "Particulate Matter" OR "$PM_{2.5}$" OR "PM_{10}" OR "Particles" OR "Gaseous air pollutant"<br>#2 "Parkinson Disease"[Mesh] OR "Parkinson Disease" OR "Idiopathic Parkinson's Disease" OR "Lewy Body Parkinson's Disease" OR "Parkinson's Disease" OR "Idiopathic Parkinson Disease" OR "Lewy Body Parkinson Disease" OR "Primary Parkinsonism" OR "Paralysis Agitans"<br>#3 "Alzheimer Disease"[Mesh] OR "Alzheimer Disease" OR "Alzheimer Syndrome" OR "Alzheimer Type Dementia(ATD)" OR "Alzheimer Type Senile Dementia" OR "Alzheimer Dementia" OR "Alzheimer Dementias" OR "Alzheimer's Disease" OR "Alzheimer's Diseases" OR "Alzheimer Diseases" OR "Alzheimers Diseases"<br>#1 AND (#2 OR #3)
**2. Web of Science (WOS)**	#1 TS=("Air Pollution" OR "Air pollutant*" OR "Air Quality" OR "Carbon monoxide" OR "Sulfur dioxide" OR "Nitrogen dioxide" OR "Ozone" OR "Gaseous air pollutant" OR "Particulate Matter" OR "$PM_{2.5}$" OR "PM_{10}" OR "Particles")<br>#2 TS=("Parkinson Disease" OR "Idiopathic Parkinson's Disease" OR "Lewy Body Parkinson's Disease" OR "Parkinson's Disease" OR "Idiopathic Parkinson Disease" OR "Lewy Body Parkinson Disease" OR "Primary Parkinsonism" OR "Paralysis Agitans")<br>#3 TS=("Alzheimer Disease" OR "Alzheimer Syndrome" OR "Alzheimer Type Dementia (ATD)" OR "Alzheimer Type Senile Dementia" OR "Alzheimer Dementia" OR "Alzheimer Dementias" OR "Alzheimer's Disease" OR "Alzheimer's Diseases" OR "Alzheimer Diseases" OR "Alzheimers Diseases")<br>#1 AND (#2 OR #3)
**3. Scopus**	1 TITLE-ABS-KEY("Air Pollution" OR "Air pollutant*" OR "Air Quality" OR "Carbon monoxide" OR "Sulfur dioxide" OR "Nitrogen dioxide" OR "Ozone" OR "Gaseous air pollutant" OR "Particulate Matter" OR "$PM_{2.5}$" OR "PM_{10}" OR "Particles")<br>2 TITLE-ABS-KEY("Parkinson Disease" OR "Idiopathic Parkinson's Disease" OR "Lewy Body Parkinson's Disease" OR "Parkinson's Disease" OR "Idiopathic Parkinson Disease" OR "Lewy Body Parkinson Disease" OR "Primary Parkinsonism" OR "Paralysis Agitans") OR TITLE-ABS-KEY("Alzheimer Disease" OR "Alzheimer Syndrome" OR "Alzheimer Type Dementia (ATD)" OR "Alzheimer Type Senile Dementia" OR "Alzheimer Dementia" OR "Alzheimer Dementias" OR "Alzheimer's Disease" OR "Alzheimer's Diseases" OR "Alzheimer Diseases" OR "Alzheimers Diseases")<br>1 AND 2
**4. EMBASE**	('air pollution'/exp OR 'air pollution' OR 'air pollutant' OR 'air quality' OR 'carbon monoxide' OR 'sulfur dioxide' OR 'nitrogen dioxide' OR 'ozone' OR 'particulate matter' OR '$PM_{2.5}$' OR 'PM_{10}' OR 'particles' OR 'gaseous air pollutant')<br>AND ('parkinson disease'/exp OR 'parkinson disease' OR 'idiopathic parkinson s disease' OR 'lewy body parkinson s disease' OR 'parkinson s disease' OR 'idiopathic parkinson disease' OR 'lewy body parkinson disease' OR 'primary parkinsonism' OR 'paralysis agitans' OR 'alzheimer disease'/exp OR 'alzheimer disease' OR 'alzheimer syndrome' OR 'alzheimer type dementia (atd)' OR 'alzheimer type senile dementia' OR 'alzheimer dementia' OR 'alzheimer dementias' OR 'alzheimer s disease' OR 'alzheimer s diseases' OR 'alzheimer diseases' OR 'alzheimers diseases')

Table S3. Explanatory file for Effective Public Health Practice Project (EPHPP) Quality Assessment Tool.

Component ratings	Details
**A. Selection bias**	Good: (Q1 is 1) and (Q2 is 1).<br>Fair: (Q1 is 1 or 2) and (Q2 is 2 or 5).<br>Poor: (Q1 is 3); or (Q2 is 3); or (Q1 is 4) and (Q2 is 5).
**Q1. Are the individuals selected to participate in the study likely to be representative of the target population?**	
1 Very likely	Randomly selected from a comprehensive list of individuals in the target population
2 Somewhat likely	Referred from a source (e.g. clinic) in a systematic manner
3 Not likely	Self-referred
4 Cannot tell	
**Q2. What percentage of selected individuals agreed to participate?**	
1 80 - 100% agreement<br>2 60 – 79% agreement<br>3 Less than 60% agreement<br>4 Not applicable<br>5 Can't tell	Refers to the percentage of subjects in the control and intervention groups that agreed to participate in the study before they were assigned to intervention or control groups.
**B. Study design**	Good: (Q1 is 1).<br>Fair: (Q1 is 2).<br>Weak: (Q1 is 3).
**Q1. Indicate the study design**	
1 Strong design	RCTs and CCTs.
2 Moderate design	Cohort , case-control or case crossover
3 Weak design	Cross-sectional or time-series
**C. Confounders**	Good: (Q1 is 2); or (Q2 is 1).<br>Fair: (Q1 is 1) and (Q2 is 2).<br>Poor: (Q1 is 1) and (Q2 is 3); or (Q1 is 3) and (Q2 is 4).
**Q1.Were there important differences between groups prior to the intervention?**	
1 Yes	
2 No	
**Q2.If yes, indicate the percentage of relevant confounders that were controlled (either in the design (e.g. stratification, matching) or analysis)?**	
1 80-100%	
2 60-79%	
3 Less than 60% or none	
**D. Blinding**	Good: (Q1 is 2) and (Q2 is 2).<br>Fair: (Q1 is 2); or (Q2 is 2).<br>Poor: (Q1 is 1) and (Q2 is 1); or (Q1 is 3) and (Q2 is 3).
Q1. Was (were) the outcome assessor(s) aware of the intervention or exposure status of participants?	
1 Yes	The outcome assessors were aware of the exposure status of participants
2 No	The outcome assessors were not aware of the exposure status of participants
**Q2. Were the study participants aware of the research question?**	

1 Yes	The participants were aware of the exposure status of participants(need for informed consent)
2 No	The participants were not aware of the exposure status of participants(waive need for informed consent)
**E. Data collection methods**	Good: (Q1 is 1) and (Q2 is 1).<br>Fair: (Q1 is 1) and (Q2 is 2).<br>Poor: (Q1 is 2).
**Q1.Were data collection tools shown to be valid?**	
1 Yes	Data extracted from surveys, hospital databases, or researches conducted by professionals and trained investigators.
2 No	Data source was not introduced.
**Q2. Were data collection tools shown to be reliable?**	
1 Yes	Especially outcome assessment. Methods had been officially acknowledged (e.g. ICD/DSM) or had evidence from previous researches.
2 No	Especially outcome assessment. Methods not widely accepted.
**F. WITHDRAWALS AND DROP-OUTS**	Good: (Q1 is 1) and (Q2 is 1).<br>Fair: (Q2 is 2).<br>Poor: (Q2 is 3); or (Q1 is 2).<br>Not Applicable: (Q1 is 3); or (Q2 is 4).
**Q1.Were withdrawals and drop-outs reported in terms of numbers and/or reasons per group?**	
1 Yes	Describe both the numbers and reasons for withdrawals and drop-outs
2 No	Either the numbers or reasons for withdrawals and drop-outs are not reported
3 Not Applicable	not follow-up data(i.e. one time surveys or interviews)
**Q2.Indicate the percentage of participants completing the study. (If the percentage differs by groups, record the lowest).**	
1 80 -100%<br>2 60 - 79%<br>3 less than 60%	The percentage of participants completing the study refers to the % of subjects remaining in the study at the final data collection period in all groups
4 Not Applicable	Not Applicable(i.e. case control)

Overall ratings:

1) STRONG - no “Poor” ratings

2) MODERATE - one “Poor” rating

3) WEAK – two or more “Poor” ratings

Table S4. Quality assessment using the Effective Public Health Practice Project (EPHPP) Quality Assessment Tool for the included studies for AD.

NO.	Reference	Selection bias	Study design	Confounders	Blinding	Data collcetion method	Withdrawals and drop-outs	Overall
1	Carey et al (2018)	Moderate	Moderate	Strong	Strong	Strong	Not Applicable	Strong
2	Cerza et al (2019)	Moderate	Moderate	Strong	Strong	Strong	Not Applicable	Strong
3	Culqui et al (2017)	Moderate	Weak	Strong	Strong	Strong	Not Applicable	Moderate
4	de Crom et al (2023)	Moderate	Moderate	Strong	Moderate	Strong	Not Applicable	Strong
5	Gandini et al (2018)	Moderate	Moderate	Strong	Strong	Strong	Not Applicable	Strong
6	Jung et al (2015)	Moderate	Moderate	Strong	Strong	Strong	Not Applicable	Strong
7	Kioumourtzoglou et al (2016)	Moderate	Moderate	Strong	Strong	Strong	Not Applicable	Strong
8	Mortamais et al (2021)	Weak	Moderate	Strong	Moderate	Strong	Not Applicable	Moderate
9	Nunez et al (2021)	Moderate	Weak	Strong	Strong	Strong	Not Applicable	Moderate
10	Ran et al (2020)	Moderate	Moderate	Strong	Moderate	Strong	Not Applicable	Strong
11	Shaffer et al (2021)	Moderate	Moderate	Strong	Moderate	Strong	Weak	Moderate
12	Shi et al (2020)	Moderate	Moderate	Strong	Strong	Strong	Not Applicable	Strong
13	Shi et al (2021)	Moderate	Moderate	Strong	Strong	Strong	Not Applicable	Strong
14	Shim et al (2023)	Moderate	Moderate	Strong	Strong	Strong	Not Applicable	Strong
15	Trevenen et al (2022)	Weak	Moderate	Strong	Strong	Strong	Not Applicable	Moderate
16	Yang et al (2022)	Weak	Moderate	Strong	Moderate	Strong	Not Applicable	Moderate
17	Yang et al (2024)	Moderate	Weak	Strong	Strong	Strong	Not Applicable	Moderate
18	Younan et al (2022)	Moderate	Moderate	Strong	Moderate	Strong	Not Applicable	Strong
19	Yuchi et al (2020)	Moderate	Moderate	Strong	Strong	Strong	Not Applicable	Strong
20	Zanobetti et al (2014)	Moderate	Moderate	Strong	Strong	Strong	Not Applicable	Strong
21	Zhang et al (2022)	Moderate	Moderate	Strong	Moderate	Strong	Not Applicable	Strong
22	Zhang et al (2023)	Moderate	Moderate	Strong	Strong	Strong	Not Applicable	Strong
23	Zhu et al (2023)	Moderate	Moderate	Strong	Moderate	Strong	Not Applicable	Strong

Abbreviation: AD, Alzheimer's disease.

Table S5. Quality assessment using the Effective Public Health Practice Project (EPHPP) Quality Assessment Tool for the included studies for PD.

NO.	Reference	Selection bias	Study design	Confounders	Blinding	Data collcetion method	Withdrawals and drop-outs	Overall
1	Cerza et al (2018)	Moderate	Moderate	Strong	Strong	Strong	Not Applicable	Strong
2	Chen et al (2017)	Moderate	Moderate	Strong	Strong	Strong	Not Applicable	Strong
3	Finkelstein，Jerrett（2007）	Moderate	Moderate	Strong	Strong	Strong	Not Applicable	Strong
4	Gandini et al (2018)	Moderate	Moderate	Strong	Strong	Strong	Not Applicable	Strong
5	Goria et al (2021)	Moderate	Weak	Strong	Strong	Strong	Not Applicable	Moderate
6	Gu et al (2020)	Moderate	Weak	Strong	Strong	Strong	Not Applicable	Moderate
7	Kioumourtzoglou et al (2016)	Moderate	Moderate	Strong	Strong	Strong	Not Applicable	Strong
8	Kirrane et al（2015）	Moderate	Moderate	Strong	Moderate	Strong	Not Applicable	Strong
9	Lee et al (2016)	Moderate	Moderate	Strong	Strong	Strong	Not Applicable	Strong
10	Lee et al (2017)	Moderate	Moderate	Strong	Strong	Strong	Not Applicable	Strong
11	Lee et al (2022)	Moderate	Moderate	Strong	Strong	Strong	Not Applicable	Strong
12	Liu et al (2016)	Moderate	Moderate	Strong	Moderate	Strong	Not Applicable	Strong
13	Nunez et al (2021)	Moderate	Weak	Strong	Strong	Strong	Not Applicable	Moderate
14	Palacios et al (2014)	Moderate	Moderate	Strong	Strong	Strong	Not Applicable	Strong
15	Palacios et al (2017)	Moderate	Moderate	Strong	Moderate	Strong	Not Applicable	Strong
16	Ritz et al (2016)	Moderate	Moderate	Strong	Moderate	Strong	Weak	Moderate
17	Rumrich et al (2023)	Moderate	Moderate	Strong	Strong	Strong	Not Applicable	Strong
18	Salimi et al (2019)	Moderate	Weak	Strong	Moderate	Strong	Not Applicable	Moderate
19	Shi et al (2020)	Moderate	Moderate	Strong	Strong	Strong	Not Applicable	Strong
20	Shin et al (2018)	Moderate	Moderate	Strong	Strong	Strong	Not Applicable	Strong
21	Toro et al (2019)	Weak	Moderate	Strong	Moderate	Strong	Not Applicable	Moderate
22	Wei et al (2019)	Moderate	Moderate	Strong	Strong	Strong	Not Applicable	Strong
23	Yu et al (2021)	Moderate	Moderate	Strong	Moderate	Strong	Not Applicable	Strong
24	Yuchi et al (2020)	Moderate	Moderate	Strong	Strong	Strong	Not Applicable	Strong
25	Zanobetti et al (2014)	Moderate	Moderate	Strong	Strong	Strong	Not Applicable	Strong

Abbreviation: PD, Parkinson’s disease.

Table S6. The detailed quantity of effect estimates included in the meta-analyses.

Outcome	Pollutant	Exposure duration		
		Long-term	Short-term	Total
AD	$PM_{2.5}$	17	4	21
	PM_{10}	5	1	6
	NO_2	10	1	11
	O_3	4	1	5
PD	$PM_{2.5}$*	17	5	22
	PM_{10}	10	1	11
	NO_2	11	2	13
	O_3*	6	3	9
	SO_2	2	1	3
	CO	3	1	4

*An US study (study of the associations between long-term exposures to $PM_{2.5}$ and O_3 and PD) reported results for two states (North Carolina/Iowa), and the estimates for both states were included simultaneously; in the main text of the meta-analysis, the number of studies is enumerated, not the number of estimates.

Abbreviations: AD, Alzheimer's disease; CO, carbon monoxide; NO_2, nitrogen dioxide; O_3, ozone; PD, Parkinson's disease; $PM_{2.5}$, particulate matter with an aerodynamic diameter of or smaller than 2.5 μm; PM_{10}, particulate matter with an aerodynamic diameter of or smaller than 10 μm; SO_2, sulfur dioxide.

Table S7. Subgroup analysis for the meta-analysis on the association between $PM_{2.5}$ exposure and risk of AD or PD.

Subgroup	Study No.	Estimate (95% CI)	P-value	I-squared	P for heterogeneity	P for subgroup difference
**Long-term $PM_{2.5}$ exposure-AD**						
Study area						
Europe	6	1.10 (0.94, 1.30)	0.227	83.09%	<0.001	0.095
Asia	4	1.02 (1.00, 1.03)	0.074	51.94%	0.1	
North America	6	1.34 (1.04, 1.73)	0.023	88.38%	<0.001	
Oceania	1	0.94 (0.78, 1.13)	0.503	-	-	
Sample size						
≥100000	6	1.31 (1.03, 1.67)	0.028	94.81%	<0.001	0.102
<100000	11	1.06 (1.00, 1.14)	0.064	61.71%	0.004	
Age*						
≥65 years	10	1.16 (0.99, 1.34)	0.062	97.08%	<0.001	0.540
<65 years	2	1.31 (1.08, 1.59)	0.006	0.00%	0.497	
Female proportion*						
≥50%	14	1.22 (1.07, 1.38)	0.002	94.94%	<0.001	0.008
<50%	2	1.01 (0.98, 1.04)	0.531	0.00%	0.435	
Study design						
Cohort	15	1.17 (1.04, 1.32)	0.009	95.67%	<0.001	0.171
Time-series	1	1.28 (0.98, 1.66)	0.067	-	-	
Case-control	1	0.71 (0.41, 1.24)	0.227	-	-	
Exposure assessment						
Model prediction	15	1.11 (1.02, 1.20)	0.016	93.75%	<0.001	0.471
Fixed site	2	1.42 (0.72, 2.78)	0.309	98.29%	<0.001	
Study quality						
Moderate	3	1.31 (1.03, 1.66)	0.029	0.00%	0.631	0.349
High	14	1.15 (1.02, 1.30)	0.027	95.99%	<0.001	
**Short-term $PM_{2.5}$ exposure-AD**						

Subgroup	Study No.	Estimate (95% CI)	P-value	I-squared	P for heterogeneity	P for subgroup difference
Study area						
Europe	1	1.08 (1.04, 1.13)	<0.001	-	-	<0.001
Asia	1	1.02 (1.01, 1.03)	0.003	-	-	
North America	2	1.00 (1.00, 1.00)	0.035	0.00%	0.715	
Sample size						
≥100000	3	1.02 (0.98, 1.07)	0.325	82.83%	0.003	0.808
<100000	1	1.02 (1.01, 1.03)	0.003	-	-	
Study design						
Time-series	2	1.05 (0.98, 1.11)	0.153	85.94%	0.008	0.715
Case-control	2	1.00 (1.00, 1.00)	0.035	0.00%	0.715	
Exposure assessment						
Fixed site	2	1.05 (0.98, 1.11)	0.153	85.94%	0.008	0.175
Model prediction	2	1.00 (1.00, 1.00)	0.035	0.00%	0.715	
Study quality						
Moderate	2	1.05 (0.98, 1.11)	0.153	85.94%	0.008	0.175
High	2	1.00 (1.00, 1.00)	0.035	0.00%	0.715	
**Long-term $PM_{2.5}$ exposure-PD**						
Study area						
Europe	4	0.98 (0.95, 1.01)	0.209	0.00%	0.424	<0.001
Asia	2	1.17 (1.07, 1.29)	0.001	2.35%	0.312	
North America	10	1.15 (1.04, 1.28)	0.006	96.91%	<0.001	
Oceania	1	1.05 (0.91, 1.22)	0.512	-	-	
Sample size						
≥100000	10	1.12 (1.03, 1.22)	0.010	92.64%	<0.001	0.333
<100000	7	1.06 (0.98, 1.15)	0.176	59.19%	0.023	
Age*						
≥65 years	5	1.12 (0.97, 1.29)	0.121	93.38%	<0.001	0.898

Subgroup	Study No.	Estimate (95% CI)	P-value	I-squared	P for heterogeneity	P for subgroup difference
<65 years	9	1.09 (1.00, 1.18)	0.039	71.03%	0.001	
Female proportion*						
≥50%	9	1.11 (1.03, 1.20)	0.009	90.36%	<0.001	0.008
<50%	8	1.00 (0.99, 1.01)	0.630	67.34%	0.003	
Study design						
Cohort	10	1.10 (1.02, 1.18)	0.008	97.15%	<0.001	<0.001
Time-series	1	1.54 (1.22, 1.94)	<0.001	-	-	
Case-control	5	0.99 (0.96, 1.03)	0.737	0.30%	0.404	
Cross-sectional	1	1.05 (0.91, 1.22)	0.512	-	-	
Exposure assessment						
Model prediction	16	1.07 (1.02, 1.13)	0.007	95.56%	<0.001	0.001
Fixed site	1	1.47 (1.22, 1.77)	<0.001	-	-	
Study quality						
Moderate	5	1.23 (0.95, 1.59)	0.111	59.38%	0.043	0.324
High	12	1.08 (1.02, 1.14)	0.011	96.73%	<0.001	
**Short-term $PM_{2.5}$ exposure-PD**						
Study area						
Europe	1	1.00 (1.00, 1.01)	0.005	-	-	0.038
Asia	2	1.02 (1.01, 1.02)	<0.001	0.00%	0.959	
North America	2	1.11 (0.88, 1.39)	0.375	85.74%	0.008	
Sample size						
≥100000	2	1.00 (1.00, 1.01)	0.005	34.12%	0.218	0.002
<100000	3	1.02 (1.01, 1.02)	<0.001	67.94%	0.044	
Study design						
Time-series	2	1.00 (1.00, 1.01)	0.005	34.12%	0.218	0.002
Case-control	3	1.02 (1.01, 1.02)	<0.001	67.94%	0.044	
Exposure assessment						

Subgroup	Study No.	Estimate (95% CI)	P-value	I-squared	P for heterogeneity	P for subgroup difference
Fixed site	3	1.00 (1.00, 1.00)	<0.001	76.46%	0.014	0.002
Model prediction	2	1.02 (1.01, 1.02)	<0.001	0.00%	0.959	
Study quality						
Moderate	2	1.00 (1.00, 1.01)	0.005	34.12%	0.218	0.002
High	3	1.02 (1.01, 1.02)	<0.001	67.94%	0.044	

*The summary number of articles for subgroup analysis was smaller than the total number due to the absence of details in some articles.

Abbreviations: AD, Alzheimer's disease; PD, Parkinson's disease; $PM_{2.5}$, particulate matter with an aerodynamic diameter of or smaller than 2.5 μm.

Table S8. Subgroup analysis for the meta-analysis on the association between NO_2 exposure and risk of AD or PD.

Subgroup	Study No.	Estimate (95% CI)	P-value	I-squared	P for heterogeneity	P for subgroup difference
			**Long-term NO_2 exposure-AD**			
Study area						
Europe	6	1.02 (0.99, 1.04)	0.178	90.29%	<0.001	0.302
Asia	1	1.02 (0.94, 1.09)	0.669	-	-	
North America	2	0.99 (0.97, 1.02)	0.633	82.59%	0.017	
Oceania	1	0.99 (0.96, 1.01)	0.231	-	-	
Sample size						
≥100000	4	1.01 (0.98, 1.05)	0.353	96.37%	0	0.352
<100000	6	1.00 (0.98, 1.01)	0.834	40.09%	0.138	
Age*						
≥65 years	4	0.99 (0.98, 1.01)	0.373	95.65%	<0.001	0.004
<65 years	2	1.03 (1.01, 1.05)	0.001	0.00%	0.704	
Female proportion*						
≥50%	8	1.01 (1.00, 1.03)	0.157	91.72%	<0.001	0.034
<50%	1	0.99 (0.96, 1.01)	0.231	-	-	
Study design						
Cohort	9	1.01 (0.99, 1.02)	0.264	90.79%	<0.001	0.025
Case-control	1	0.98 (0.96, 1.00)	0.049	-	-	
Study quality						
Moderate	1	1.04 (0.98, 1.12)	0.215	-	-	0.275
High	9	1.00 (0.99, 1.02)	0.579	91.22%	<0.001	
			**Long-term NO_2 exposure-PD**			
Study area						
Europe	5	1.02 (0.99, 1.05)	0.23	78.15%	0.001	0.793
NorthAmerica	3	1.01 (1.00, 1.01)	0.029	46.94%	0.152	
Asia	2	1.01 (0.99, 1.03)	0.181	0.00%	0.532	
Oceania	1	1.01 (0.99, 1.03)	0.233	-	-	

Subgroup	Study No.	Estimate (95% CI)	P-value	I-squared	P for heterogeneity	P for subgroup difference
Sample size						
≥100000	5	1.01 (1.00, 1.02)	0.218	88.39%	<0.001	0.505
<100000	6	1.01 (1.00, 1.03)	0.101	47.56%	0.090	
Age*						
≥65 years	2	1.00 (0.99, 1.02)	0.672	7.69%	0.298	0.490
<65 years	6	1.01 (1.00, 1.02)	0.151	85.32%	<0.001	
Female proportion*						
≥50%	7	1.01 (1.00, 1.01)	0.131	82.00%	<0.001	0.148
<50%	3	1.01 (0.97, 1.06)	0.583	75.81%	0.016	
Study design						
Cohort	5	1.01 (1.00, 1.02)	0.296	86.86%	<0.001	0.575
Case-control	5	1.02 (0.99, 1.05)	0.171	67.29%	0.016	
Cross-sectional	1	1.01 (0.99, 1.03)	0.233	-	-	
Exposure assessment						
Model prediction	10	1.01 (1.00, 1.02)	0.063	80.57%	<0.001	0.896
Fixed Site	1	1.01 (0.99, 1.03)	0.325	-	-	
Study quality						
High	8	1.01 (1.00, 1.01)	0.133	80.97%	<0.001	0.582
Moderate	3	1.02 (0.97, 1.06)	0.415	68.36%	0.042	

*The summary number of articles for subgroup analysis was smaller than the total number due to the absence of details in some literature.

Abbreviations: AD, Alzheimer's disease; NO_2, nitrogen dioxide; PD, Parkinson's disease.

Table S9. Publication bias assessment for the associations between ambient air pollutants and risk of PD or AD when the number of studies reached above five.

Outcome	Pollutant	Exposure duration	No. of estimates	I-squared of heterogeneity	*P* for heterogeneity	*P* for Begg's test	*P* for Egger's test
AD	$PM_{2.5}$	Long-term	17	0.95	< 0.001	0.217	0.517
	NO_2	Long-term	10	0.90	< 0.001	0.929	0.750
PD	$PM_{2.5}$	Long-term	17	0.95	< 0.001	0.070	0.788
	**PM_{10}**	Long-term	10	0.50	0.036	**0.325**	**0.036**
	**NO_2**	Long-term	11	0.79	< 0.001	**0.815**	**0.028**
	O_3	Long-term	6	0.11	0.347	0.851	0.806

Abbreviations: AD, Alzheimer's disease; NO_2, nitrogen dioxide; O_3, ozone; PD, Parkinson's disease; $PM_{2.5}$, particulate matter with an aerodynamic diameter of or smaller than 2.5 μm; PM_{10}, particulate matter with an aerodynamic diameter of or smaller than 10 μm.

Table S10. Sensitivity analyses for the meta-analysis.

Outcome	Pollutant	Metric	Study	OR (95%CI)	P-value	I-squared
AD	$PM_{2.5}$	Long-term	Omitting Carey et al(2018)	1.14 (1.02, 1.28)	0.020	95.40%
			Omitting Cerza et al(2019)	1.19 (1.06, 1.33)	0.004	94.70%
			Omitting de Crom et al(2023)	1.16 (1.04, 1.30)	0.009	95.40%
			Omitting Gandini et al(2018)	1.18 (1.04, 1.33)	0.009	95.30%
			Omitting Jung et al(2015)	1.18 (1.04, 1.33)	0.009	94.30%
			Omitting Kioumourtzoglou et al(2016)	1.09 (1.02, 1.17)	0.018	94.70%
			Omitting Mortamais et al(2021)	1.16 (1.03, 1.31)	0.018	95.40%
			Omitting Nunez et al(2021)	1.16 (1.03, 1.30)	0.018	95.40%
			Omitting Ran et al(2020)	1.17 (1.04, 1.33)	0.010	95.40%
			Omitting Shaffer et al(2021)	1.15 (1.03, 1.29)	0.015	95.40%
			Omitting Shi et al(2020)	1.16 (1.03, 1.32)	0.017	85.90%
			Omitting Trevenen et al(2022)	1.18 (1.05, 1.33)	0.006	95.40%
			Omitting Yang et al(2022)	1.18 (1.04, 1.33)	0.009	92.80%
			Omitting Younan et al(2022)	1.15 (1.02, 1.29)	0.019	95.40%
			Omitting Yuchi et al(2020)	1.18 (1.05, 1.32)	0.005	95.40%
			Omitting Zhang et al(2022)	1.16 (1.03, 1.31)	0.015	95.40%
			Omitting Zhu et al(2023)	1.15 (1.02, 1.29)	0.021	95.40%
			Pooled estimate	1.16 (1.04, 1.30)	0.010	95.10%
AD	$PM_{2.5}$	Short-term	Omitting Culqui et al(2017)	1.01 (1.00, 1.01)	0.202	69.30%
			Omitting Yang et al(2024)	1.02 (0.98, 1.07)	0.325	82.80%
			Omitting Zanobetti et al(2014)	1.03 (0.99, 1.07)	0.183	88.60%
			Omitting Zhang et al(2023)	1.03 (0.99, 1.07)	0.197	87.40%
			Pooled estimate	1.02 (0.99, 1.05)	0.185	83.20%
PD	$PM_{2.5}$	Long-term	Omitting Cerza et al(2018)	1.11 (1.04, 1.18)	0.001	95.30%
			Omitting Gandini et al(2018)	1.11 (1.03, 1.19)	0.003	95.70%
			Omitting Kioumourtzoglou et al(2016)	1.07 (1.02, 1.13)	0.007	95.60%

Outcome	Pollutant	Metric	Study	OR (95%CI)	P-value	I-squared
			Omitting Kirrane et al(2015)IA	1.09 (1.03, 1.16)	0.005	95.70%
			Omitting Kirrane et al(2015)NC	1.10 (1.03, 1.17)	0.003	95.70%
			Omitting Lee et al(2022)	1.10 (1.03, 1.18)	0.006	95.70%
			Omitting Liu et al(2016)	1.11 (1.03, 1.18)	0.003	95.70%
			Omitting Nunez et al(2021)	1.08 (1.02, 1.14)	0.006	95.60%
			Omitting Palacios et al(2014)	1.11 (1.03, 1.18)	0.004	95.70%
			Omitting Palacios et al(2017)	1.11 (1.04, 1.19)	0.002	88.80%
			Omitting Rumrich et al(2023)	1.11 (1.04, 1.19)	0.002	95.40%
			Omitting Salimi et al(2019)	1.10 (1.03, 1.18)	0.004	95.70%
			Omitting Shi et al(2020)	1.10 (1.03, 1.18)	0.008	76.90%
			Omitting Shin et al(2018)	1.11 (1.03, 1.19)	0.004	95.70%
			Omitting Toro et al(2019)	1.10 (1.03, 1.17)	0.003	95.70%
			Omitting Yu et al(2021)	1.09 (1.02, 1.16)	0.008	95.60%
			Omitting Yuchi et al(2020)	1.09 (1.02, 1.16)	0.007	95.70%
			Pooled estimate	1.10 (1.03, 1.17)	0.003	95.40%
PD	$PM_{2.5}$	Short-term	Omitting Goria et al(2021)	1.01 (1.00, 1.02)	0.037	82.30%
			Omitting Gu et al(2020)	1.01 (1.00, 1.02)	0.009	76.20%
			Omitting Lee et al(2017)	1.01 (1.00, 1.01)	0.018	72.20%
			Omitting Wei et al(2019)	1.01 (1.00, 1.01)	0.056	78.60%
			Omitting Zanobetti et al(2014)	1.01 (1.00, 1.01)	0.052	75.80%
			Pooled estimate	1.01 (1.00, 1.01)	0.016	77.40%
AD	PM_{10}	Long-term	Omitting Cerza et al(2019)	1.07 (0.97, 1.18)	0.195	61.50%
			Omitting de Crom et al(2023)	1.03 (0.96, 1.10)	0.460	72.40%
			Omitting Shim et al(2023)	1.07 (0.95, 1.19)	0.272	69.70%
			Omitting Zhang et al(2022)	0.99 (0.97, 1.01)	0.434	61.50%
			Omitting Zhu et al(2023)	0.99 (0.97, 1.01)	0.289	60.70%
			Pooled estimate	1.03 (0.96, 1.10)	0.411	64.90%
PD	PM_{10}	Long-term	Omitting Cerza et al(2018)	1.00 (0.99, 1.01)	0.999	55.50%

Outcome	Pollutant	Metric	Study	OR (95%CI)	P-value	I-squared
			Omitting Chen et al(2017)	0.99 (0.98, 1.00)	0.035	35.00%
			Omitting Lee et al(2016)	1.00 (0.99, 1.00)	0.325	28.60%
			Omitting Lee et al(2022)	0.99 (0.98, 1.00)	0.130	50.90%
			Omitting Liu et al(2016)	0.99 (0.98, 1.00)	0.114	51.30%
			Omitting Palacios et al(2014)	0.99 (0.99, 1.00)	0.250	55.40%
			Omitting Palacios et al(2017)	1.00 (0.98, 1.02)	0.976	52.10%
			Omitting Rumrich et al(2023)	1.00 (0.99, 1.01)	0.909	55.00%
			Omitting Toro et al(2019)	0.99 (0.99, 1.00)	0.272	54.90%
			Omitting Yu et al(2021)	0.99 (0.98, 1.00)	0.120	42.00%
			Pooled estimate	0.99 (0.99, 1.00)	0.235	49.90%
AD	NO_2	Long-term	Omitting Carey et al(2018)	1.00 (0.99, 1.01)	0.855	90.70%
			Omitting Cerza et al(2019)	1.01 (0.99, 1.02)	0.214	68.70%
			Omitting de Crom et al(2023)	1.00 (0.99, 1.02)	0.579	91.20%
			Omitting Gandini et al(2018)	1.01 (0.99, 1.02)	0.536	91.30%
			Omitting Mortamais et al(2021)	1.01 (0.99, 1.02)	0.461	91.30%
			Omitting Shi et al(2021)	1.01 (0.99, 1.02)	0.463	84.90%
			Omitting Trevenen et al(2022)	1.01 (0.99, 1.02)	0.315	91.10%
			Omitting Yuchi et al(2020)	1.01 (0.99, 1.02)	0.264	90.80%
			Omitting Zhang et al(2022)	1.00 (0.99, 1.02)	0.846	90.40%
			Omitting Zhu et al(2023)	1.01 (0.99, 1.02)	0.488	91.30%
			Pooled estimate	1.01 (0.99, 1.02)	0.455	90.30%
PD	NO_2	Long-term	Omitting Cerza et al(2018)	1.01 (1.00, 1.01)	0.001	44.40%
			Omitting Chen et al(2017)	1.01 (1.00, 1.02)	0.063	80.60%
			Omitting Finkelstein，Jerrett(2007)	1.01 (1.00, 1.01)	0.080	78.80%
			Omitting Gandini et al(2018)	1.01 (1.00, 1.02)	0.067	80.50%
			Omitting Liu et al(2016)	1.01 (1.00, 1.02)	0.038	81.00%
			Omitting Ritz et al(2016)	1.01 (1.00, 1.01)	0.105	77.00%
			Omitting Salimi et al(2019)	1.01 (1.00, 1.02)	0.070	80.40%

Outcome	Pollutant	Metric	Study	OR (95%CI)	P-value	I-squared
			Omitting Shin et al(2018)	1.01 (1.00, 1.02)	0.050	79.00%
			Omitting Toro et al(2019)	1.01 (1.00, 1.02)	0.034	80.70%
			Omitting Yu et al(2021)	1.01 (1.00, 1.02)	0.062	80.50%
			Omitting Yuchi et al(2020)	1.01 (1.00, 1.02)	0.098	73.50%
			Pooled estimate	1.01 (1.00, 1.02)	0.045	78.90%
PD	NO_2	Short-term	Omitting Goria et al(2021)	1.09 (1.03, 1.15)	0.001	NA
			Omitting Lee et al(2017)	1.002 (1.001, 1.003)	< 0.001	NA
			Pooled estimate	1.04 (0.96, 1.13)	0.339	89.60%
AD	O_3	Long-term	Omitting Carey et al(2018)	1.00 (1.00, 1.01)	0.793	63.20%
			Omitting Cerza et al(2019)	0.98 (0.93, 1.03)	0.440	84.40%
			Omitting Jung et al(2015)	0.98 (0.93, 1.03)	0.342	79.30%
			Omitting Shi et al(2021)	0.98 (0.93, 1.03)	0.402	85.10%
			Pooled estimate	1.00 (0.99, 1.01)	0.954	78.60%
PD	O_3	Long-term	Omitting Cerza et al(2018)	1.00 (1.00, 1.01)	0.245	26.60%
			Omitting Chen et al(2017)	1.00 (1.00, 1.01)	0.004	26.70%
			Omitting Kirrane et al(2015)IA	1.00 (1.00, 1.01)	0.068	0.00%
			Omitting Kirrane et al(2015)NC	1.00 (1.00, 1.01)	0.085	7.30%
			Omitting Lee et al(2016)	1.00 (1.00, 1.01)	0.004	13.10%
			Omitting Shin et al(2018)	1.00 (1.00, 1.01)	0.299	27.80%
			Pooled estimate	1.00 (1.00, 1.01)	0.065	10.70%
PD	O_3	Short-term	Omitting Goria et al(2021)	1.00 (0.99, 1.01)	0.776	15.80%
			Omitting Gu et al(2020)	1.003 (1.002, 1.004)	< 0.001	0.00%
			Omitting Lee et al(2017)	1.00 (1.00, 1.00)	0.334	94.30%
			Pooled estimate	1.00 (1.00, 1.00)	0.284	89.20%
PD	SO_2	Long-term	Omitting Chen et al(2017)	0.96 (0.95, 0.97)	< 0.001	NA
			Omitting Lee et al(2016)	1.02 (0.99, 1.05)	0.187	NA
			Pooled estimate	0.99 (0.93, 1.05)	0.697	92.70%
PD	CO	Long-term	Omitting Chen et al(2017)	1.52 (0.75, 3.08)	0.245	89.90%

Outcome	Pollutant	Metric	Study	OR (95%CI)	P-value	I-squared
			Omitting Lee et al(2016)	1.49 (0.66, 3.34)	0.333	86.30%
			Omitting Ritz et al(2016)	1.09 (1.00, 1.19)	0.053	0.00%
			Pooled estimate	1.32 (0.82, 2.11)	0.255	80.60%

Abbreviations: AD, Alzheimer's disease; CO, carbon monoxide; NO_2, nitrogen dioxide; OR, odd ratio; O_3, ozone; PD, Parkinson's disease; $PM_{2.5}$, particulate matter with an aerodynamic diameter of or smaller than 2.5 μm; PM_{10}, particulate matter with an aerodynamic diameter of or smaller than 10 μm; SO_2, sulfur dioxide.

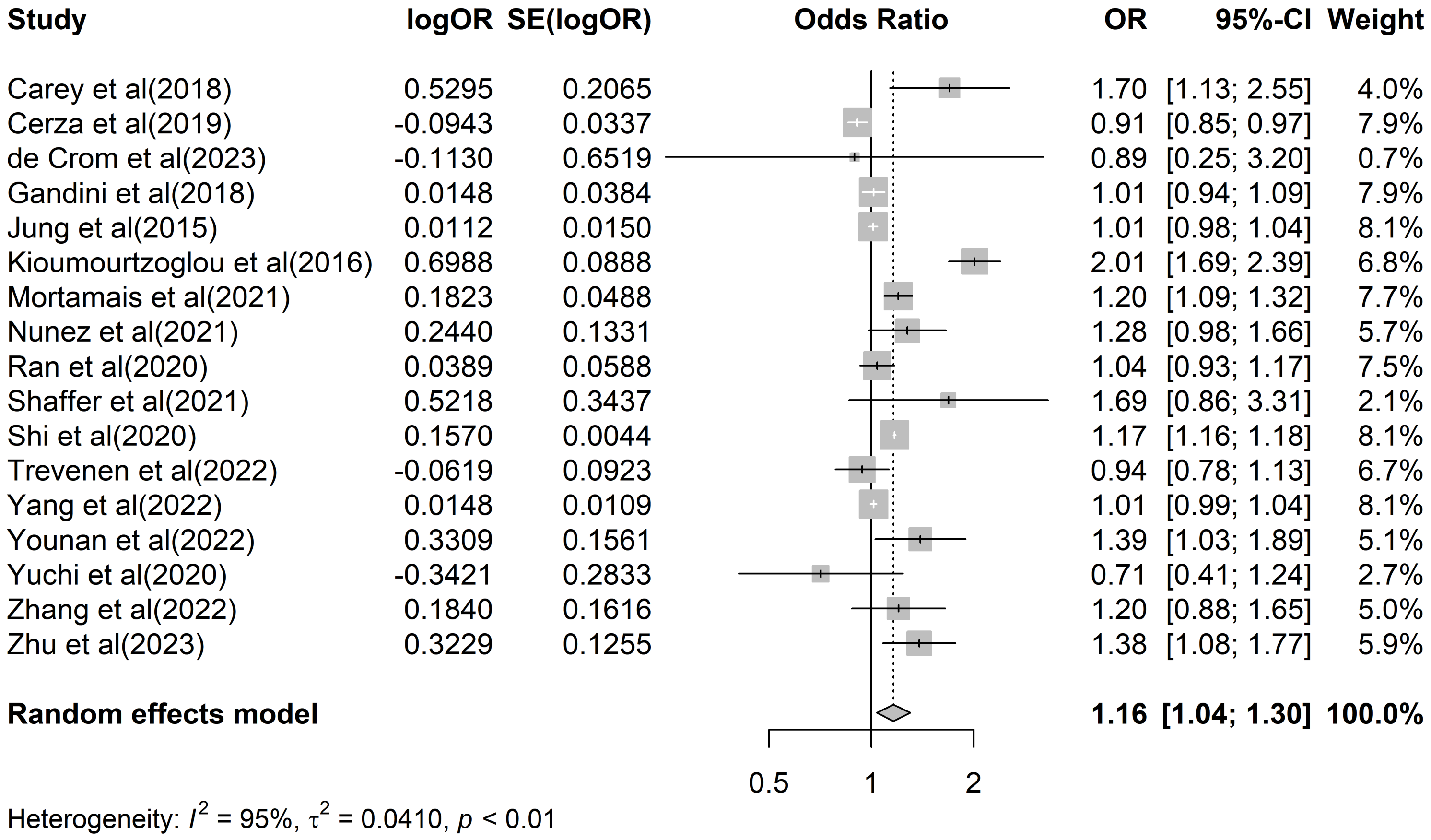

Figure S1. Forest plot for the meta-analysis on the association between long-term $PM_{2.5}$ exposure (per 5 μg/m^3 increase) and risk of AD.

Abbreviations: AD, Alzheimer's disease; OR, odds ratio; $PM_{2.5}$, particulate matter with an aerodynamic diameter of or smaller than 2.5 μm.

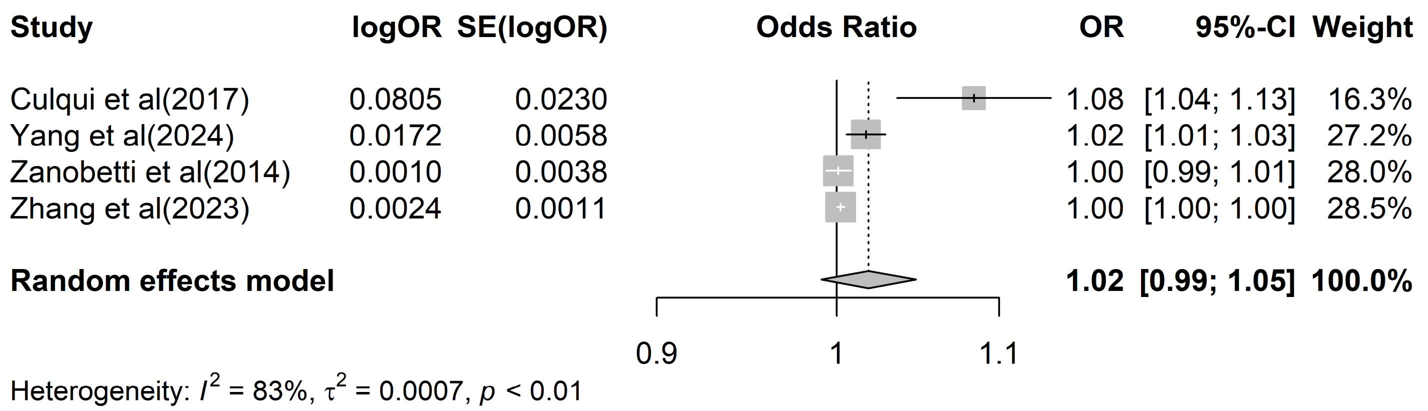

Figure S2. Forest plot for the meta-analysis on the association between short-term $PM_{2.5}$ exposure (per 5 μg/m^3 increase) and risk of AD.

Abbreviations: AD, Alzheimer's disease; OR, odds ratio; $PM_{2.5}$, particulate matter with an aerodynamic diameter of or smaller than 2.5 μm.

Study	logOR	SE(logOR)	OR	95%-CI	Weight
Cerza et al(2018)	-0.0408	0.0213	0.96	[0.92; 1.00]	8.7%
Gandini et al(2018)	0.0291	0.0384	1.03	[0.95; 1.11]	8.0%
Kioumourtzoglou et al(2016)	0.3848	0.0945	1.47	[1.22; 1.77]	5.0%
Kirrane et al(2015)IA	0.3509	0.2233	1.42	[0.92; 2.20]	1.6%
Kirrane et al(2015)NC	-0.6642	0.7043	0.51	[0.13; 2.05]	0.2%
Lee et al(2022)	0.1166	0.0634	1.12	[0.99; 1.27]	6.6%
Liu et al(2016)	0.0261	0.0528	1.03	[0.93; 1.14]	7.2%
Nunez et al(2021)	0.4309	0.1171	1.54	[1.22; 1.94]	4.0%
Palacios et al(2014)	0.0385	0.0743	1.04	[0.90; 1.20]	6.0%
Palacios et al(2017)	-0.0050	0.0064	0.99	[0.98; 1.01]	9.0%
Rumrich et al(2023)	-0.0129	0.0198	0.99	[0.95; 1.03]	8.7%
Salimi et al(2019)	0.0498	0.0758	1.05	[0.91; 1.22]	5.9%
Shi et al(2020)	0.1222	0.0045	1.13	[1.12; 1.14]	9.0%
Shin et al(2018)	0.0516	0.0225	1.05	[1.01; 1.10]	8.6%
Toro et al(2019)	-0.2231	0.7049	0.80	[0.20; 3.18]	0.2%
Yu et al(2021)	0.2098	0.0667	1.23	[1.08; 1.41]	6.4%
Yuchi et al(2020)	0.2611	0.0994	1.30	[1.07; 1.58]	4.8%
**Random effects model**			**1.10**	**[1.03; 1.17]**	**100.0%**

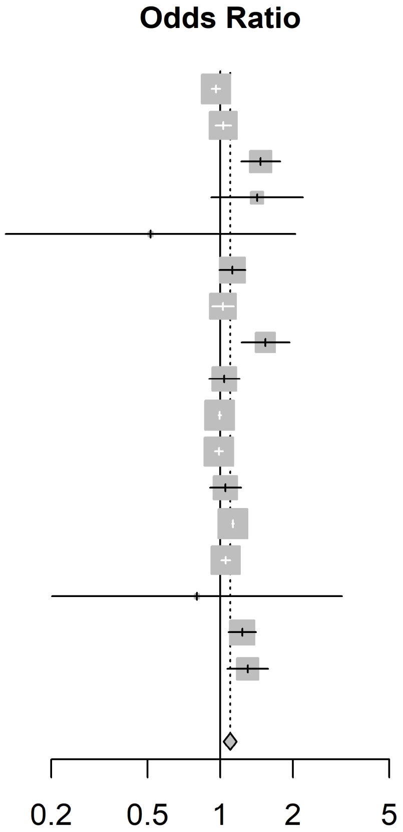

Heterogeneity: $I^2 = 95\%$, $\tau^2 = 0.0111$, $p < 0.01$

Figure S3. Forest plot for the meta-analysis on the association between long-term $PM_{2.5}$ exposure (per 5 μg/m^3 increase) and risk of PD.

Abbreviations: OR, odds ratio; PD, Parkinson’s disease; $PM_{2.5}$, particulate matter with an aerodynamic diameter of or smaller than 2.5 μm.

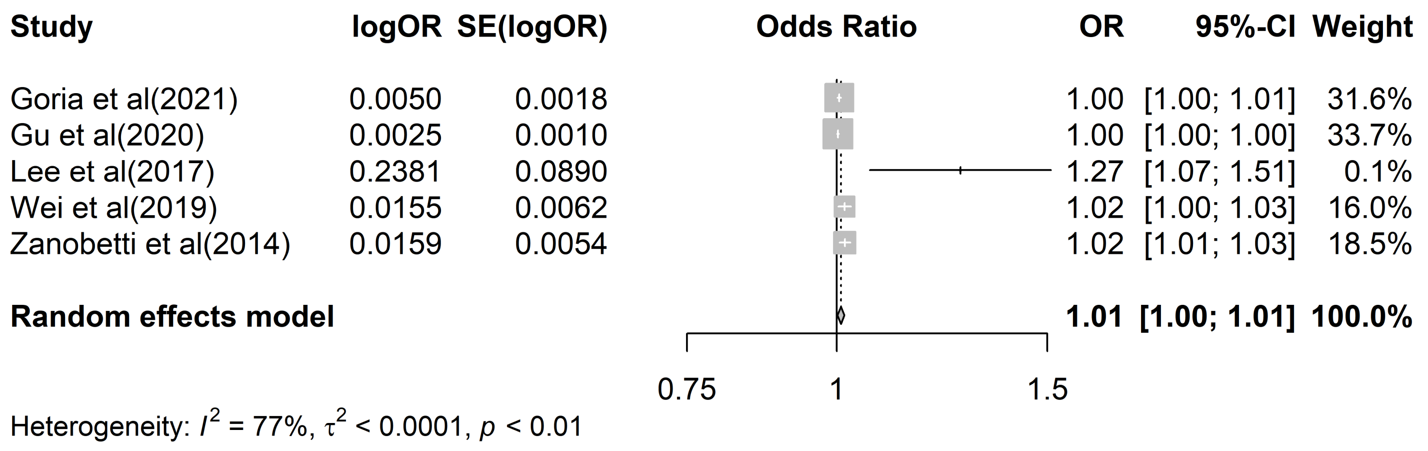

Figure S4. Forest plot for the meta-analysis on the association between short-term $PM_{2.5}$ exposure (per 5 μg/m^3 increase) and risk of PD.

Abbreviations: OR, odds ratio; PD, Parkinson’s disease; $PM_{2.5}$, particulate matter with an aerodynamic diameter of or smaller than 2.5 μm.

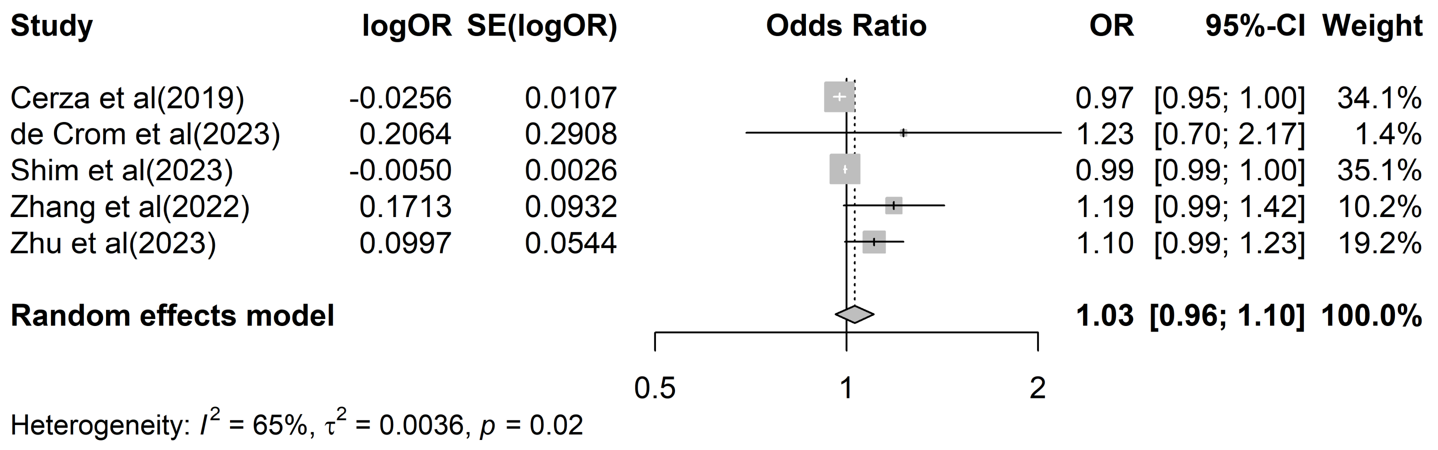

Figure S5. Forest plot for the meta-analysis on the association between long-term PM_{10} exposure (per 5 μg/m^3 increase) and risk of AD.

Abbreviations: AD, Alzheimer's disease; OR, odds ratio; PM_{10}, particulate matter with an aerodynamic diameter of or smaller than 10 μm.

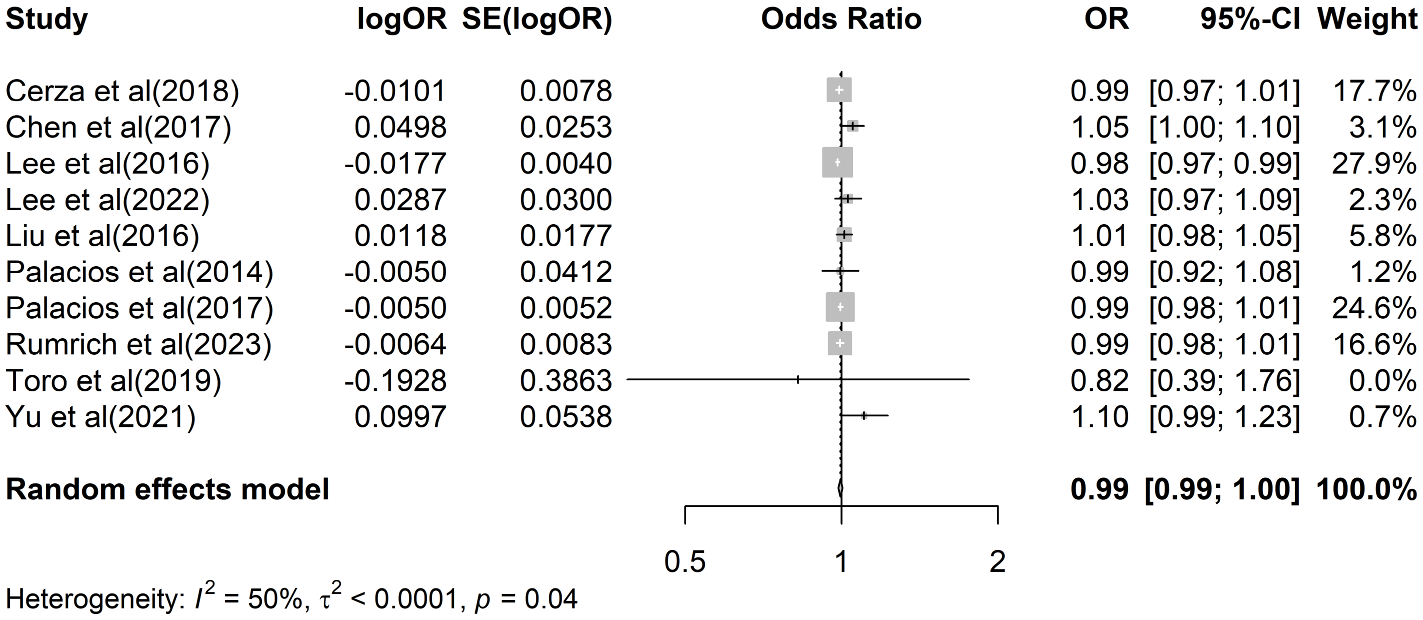

Figure S6. Forest plot for the meta-analysis on the association between long-term PM_{10} exposure (per 5 μg/m^3 increase) and risk of PD.
Abbreviations: OR, odds ratio;PD, Parkinson's disease; PM_{10}, particulate matter with an aerodynamic diameter of or smaller than 10 μm.

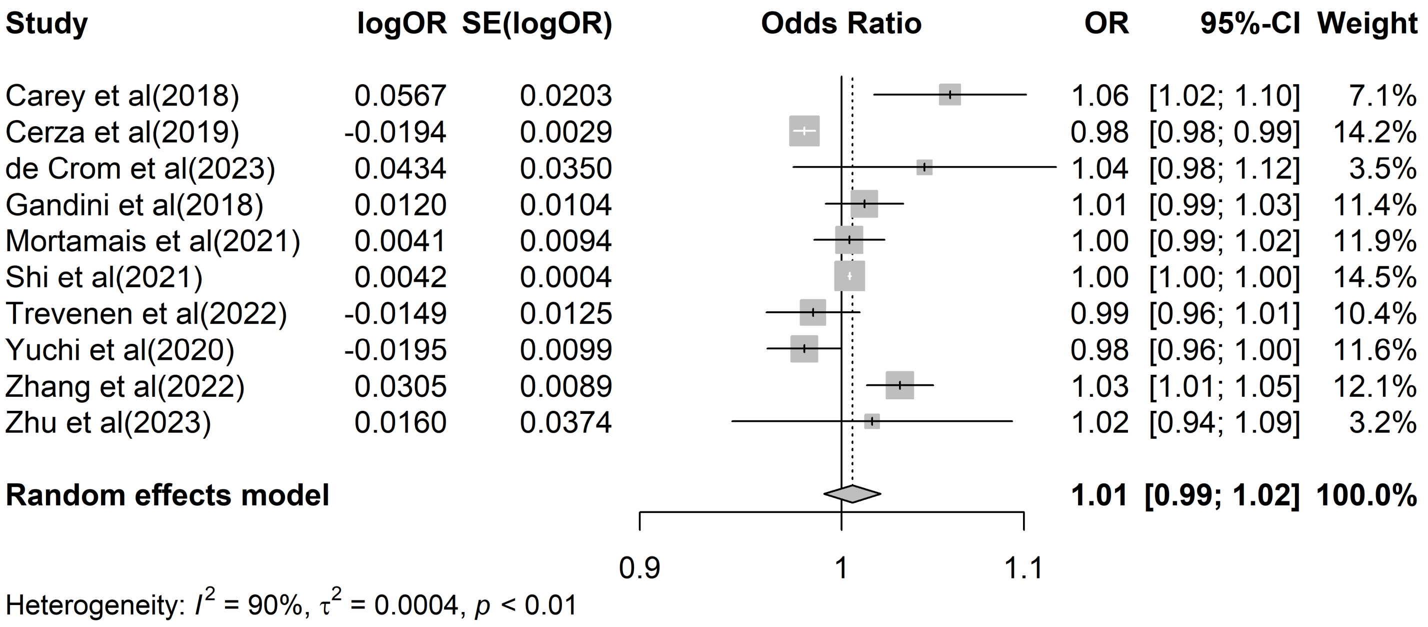

Figure S7. Forest plot for the meta-analysis on the association between long-term NO_2 exposure (per 1ppb increase)and risk of AD.

Abbreviations: AD, Alzheimer's disease; NO_2, nitrogen dioxide; OR, odds ratio.

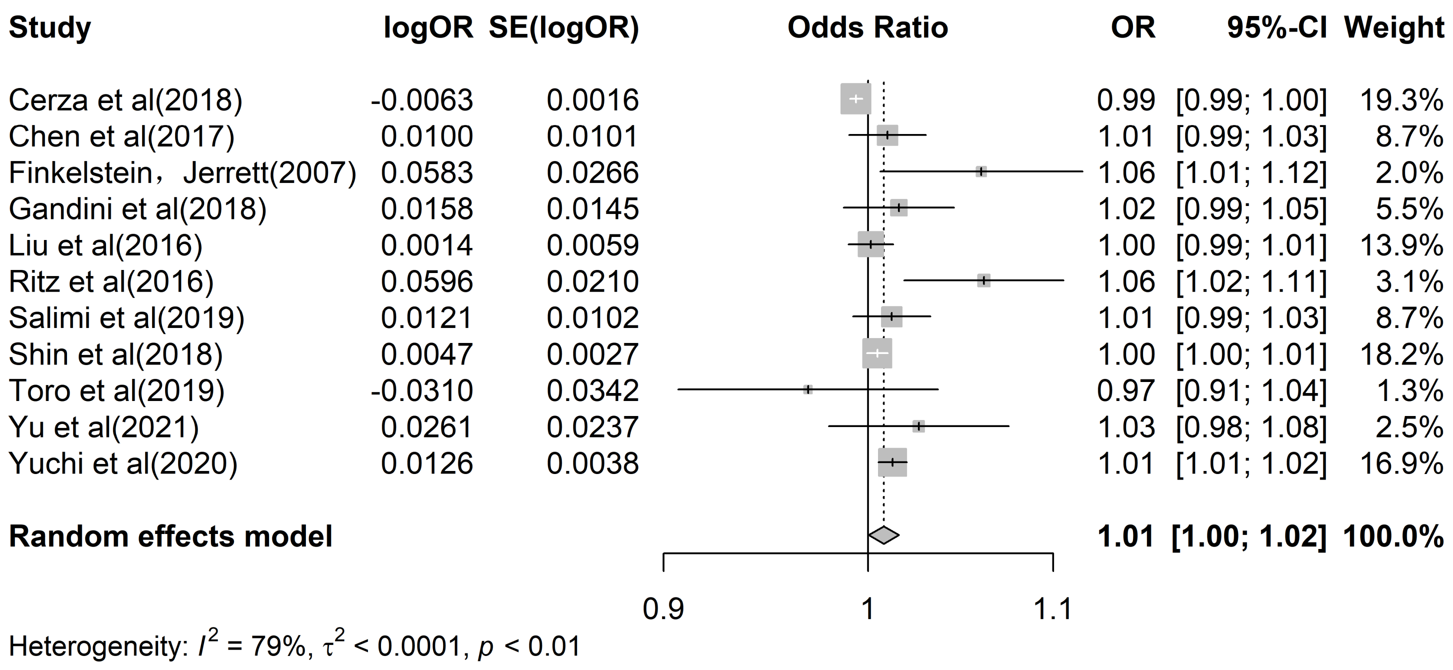

Figure S8. Forest plot for the meta-analysis on the association between long-term NO_2 exposure (per 1 ppb increase) and risk of PD.

Abbreviations: NO_2, nitrogen dioxide; OR, odds ratio; PD, Parkinson's disease.

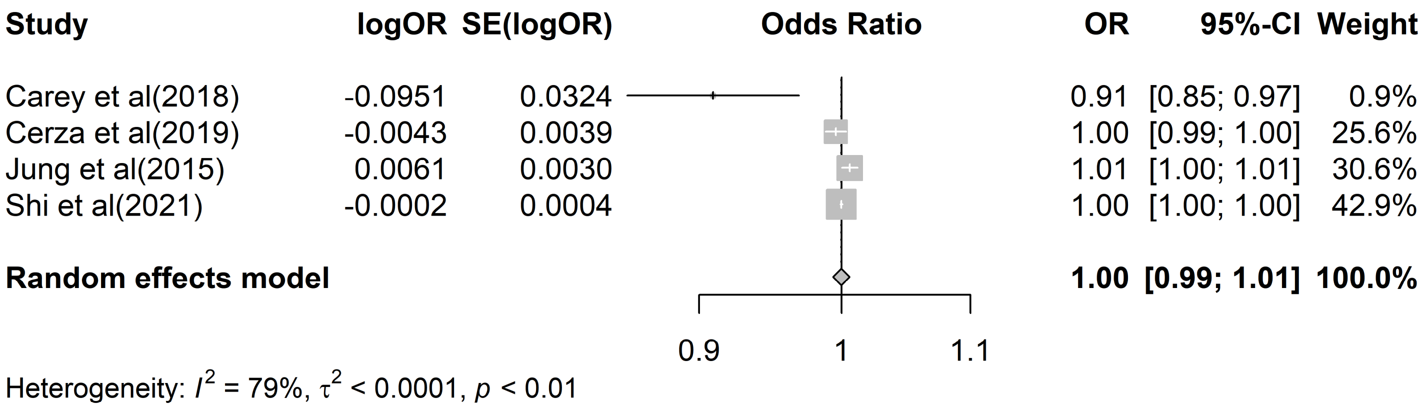

Figure S9. Forest plot for the meta-analysis on the association between long-term O_3 exposure (per 1ppb increase) and risk of AD.

Abbreviations: AD, Alzheimer's disease; OR, odds ratio; O_3, ozone.

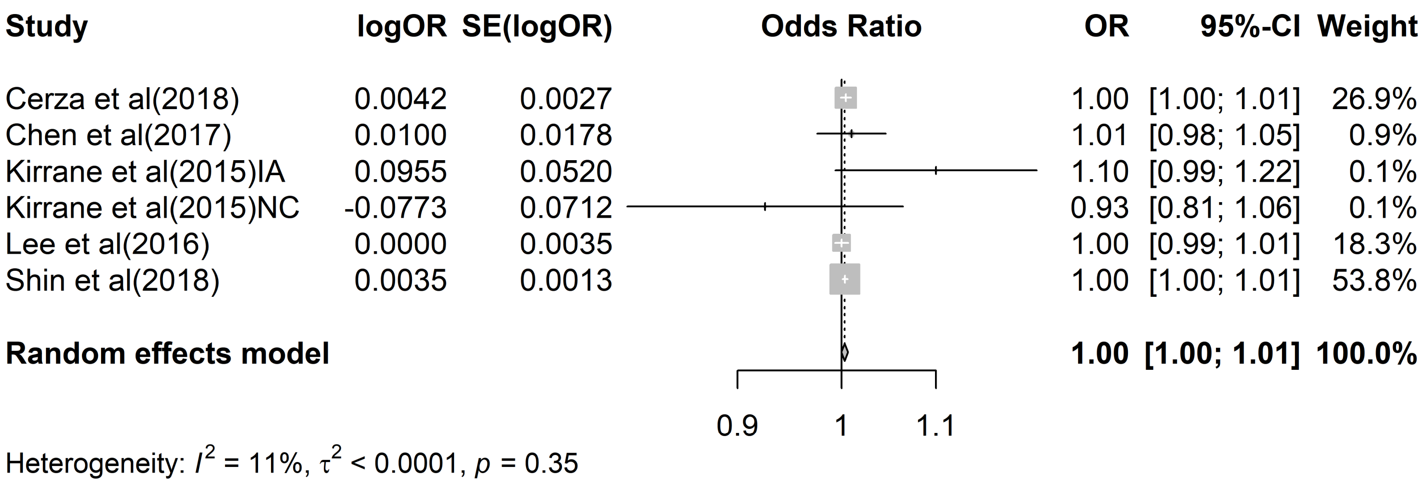

Figure S10. Forest plot for the meta-analysis on the association between long-term O_3 exposure (per 1ppb increase) and risk of PD.

Abbreviations: OR, odds ratio; O_3, ozone; PD, Parkinson's disease.

Study	logOR	SE(logOR)	OR	95%-CI	Weight
Goria et al(2021)	0.0030	0.0005	1.00	[1.00; 1.00]	49.1%
Gu et al(2020)	-0.0000	0.0005	1.00	[1.00; 1.00]	49.8%
Lee et al(2017)	0.0157	0.0144	1.02	[0.99; 1.04]	1.1%
**Random effects model**			**1.00**	**[1.00; 1.00]**	**100.0%**

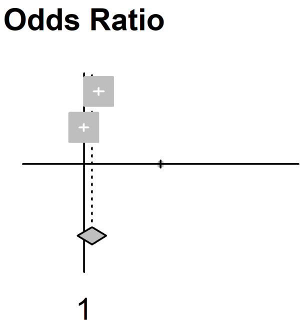

Heterogeneity: $I^2 = 89\%$, $\tau^2 < 0.0001$, $p < 0.01$

Figure S11. Forest plot for the meta-analysis on the association between short-term O_3 exposure (per 1ppb increase) and risk of PD.

Abbreviations: OR, odds ratio; O_3, ozone; PD, Parkinson's disease.

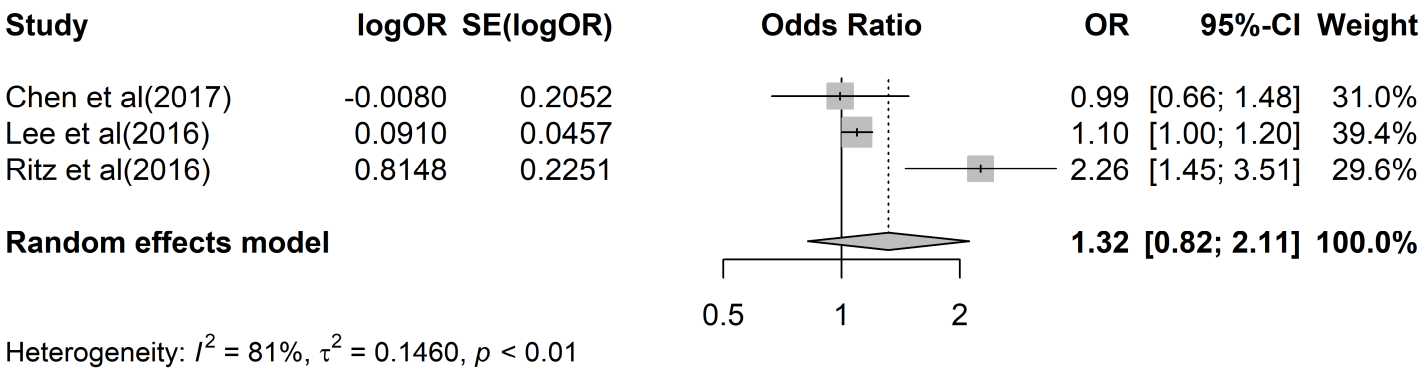

Figure S12. Forest plot for the meta-analysis on the association between long-term CO exposure (per 1mg/m^3 increase) and risk of PD.

Abbreviations: CO, carbon monoxide; OR, odds ratio; PD, Parkinson's disease.

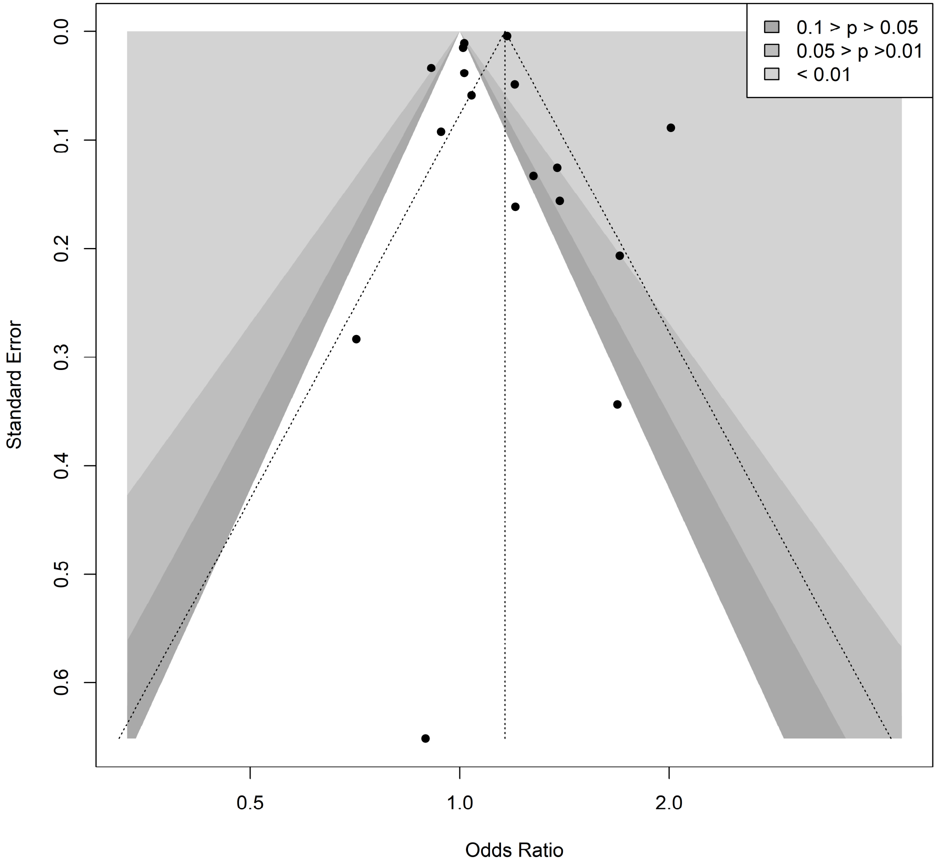

Figure S13. Funnel plot for the meta-analysis on the associations between long-term $PM_{2.5}$ exposure (per 5 μg/m^3 increase) and risk of AD.

Abbreviations: AD, Alzheimer's disease; OR, odds ratio; $PM_{2.5}$, particulate matter with an aerodynamic diameter of or smaller than 2.5 μm.

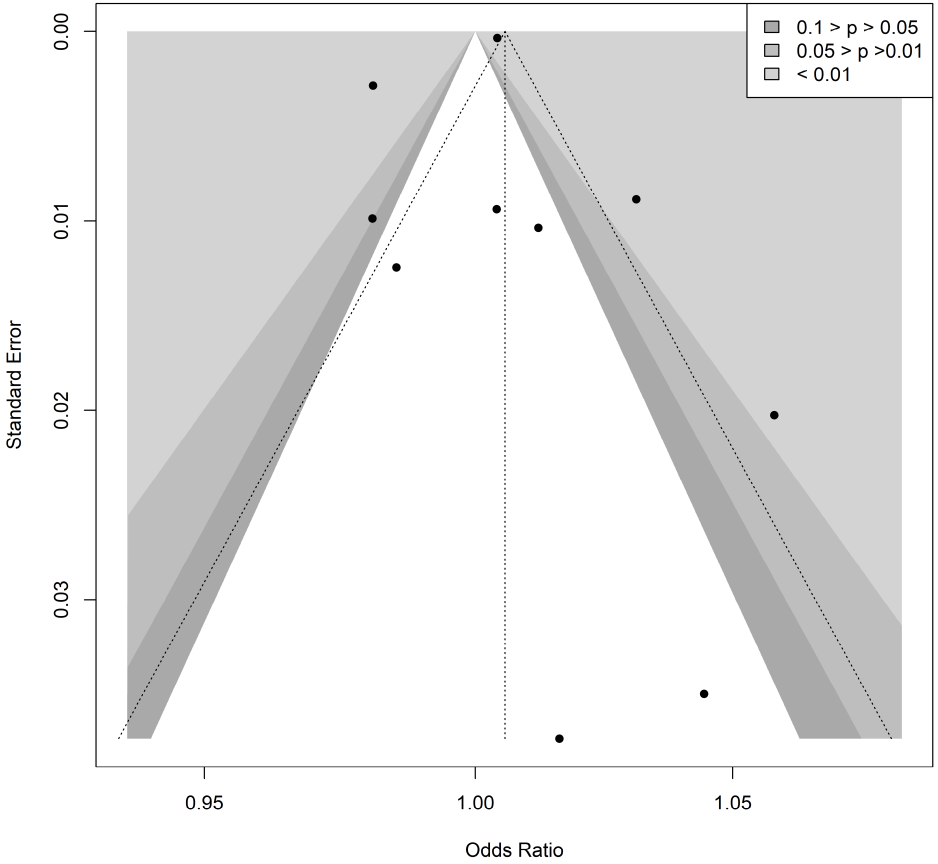

Figure S14. Funnel plot for the meta-analysis on the associations between long-term NO_2 exposure (per 1ppb increase) and risk of AD.
Abbreviations: AD, Alzheimer's disease; NO_2, nitrogen dioxide; OR, odds ratio.

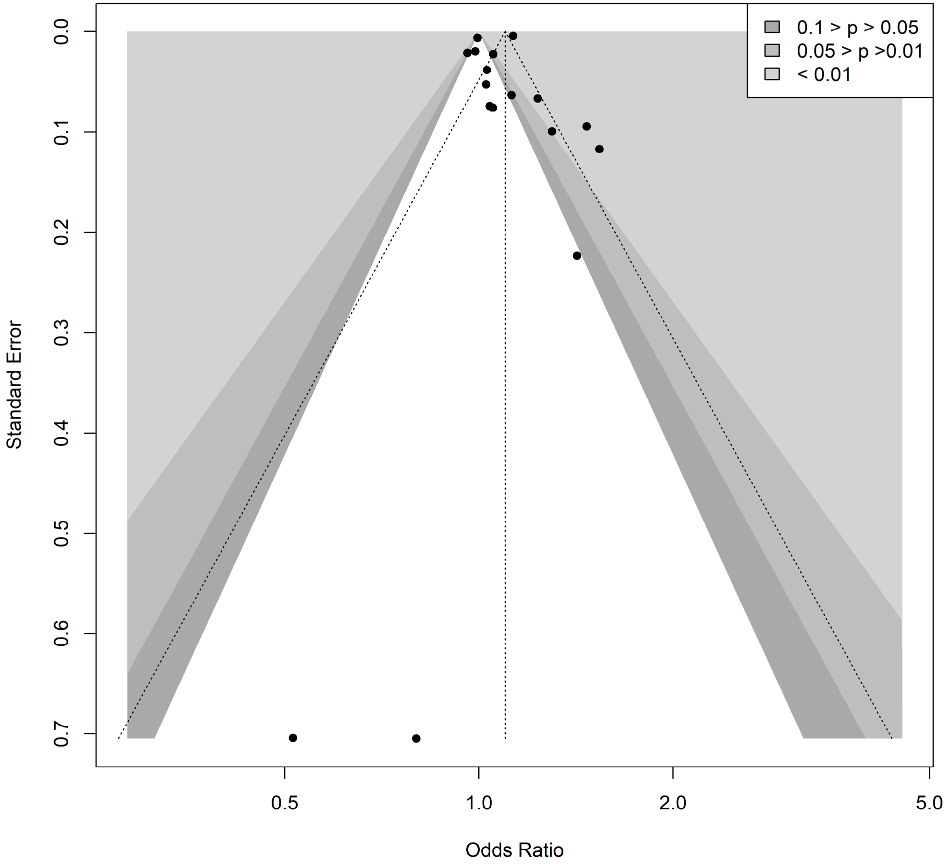

Figure S15. Funnel plot for the meta-analysis on the associations between long-term $PM_{2.5}$ exposure (per 5 μg/m^3 increase) and risk of PD.
Abbreviations: OR, odds ratio; PD, Parkinson's disease; $PM_{2.5}$, particulate matter with an aerodynamic diameter of or smaller than 2.5 μm.

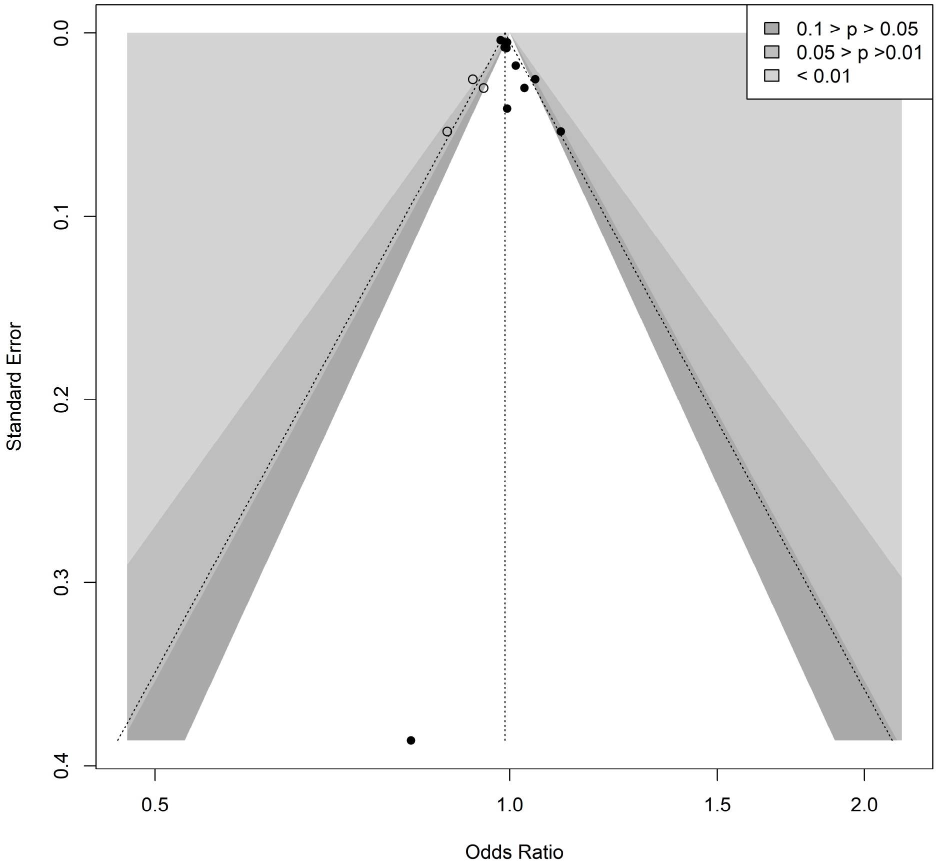

Figure S16. Funnel plot for the meta-analysis on the associations between long-term PM_{10} exposure (per 5 μg/m^3 increase) and risk of PD.

Abbreviations: OR, odds ratio; PD, Parkinson's disease; PM_{10}, particulate matter with an aerodynamic diameter of or smaller than 10 μm.

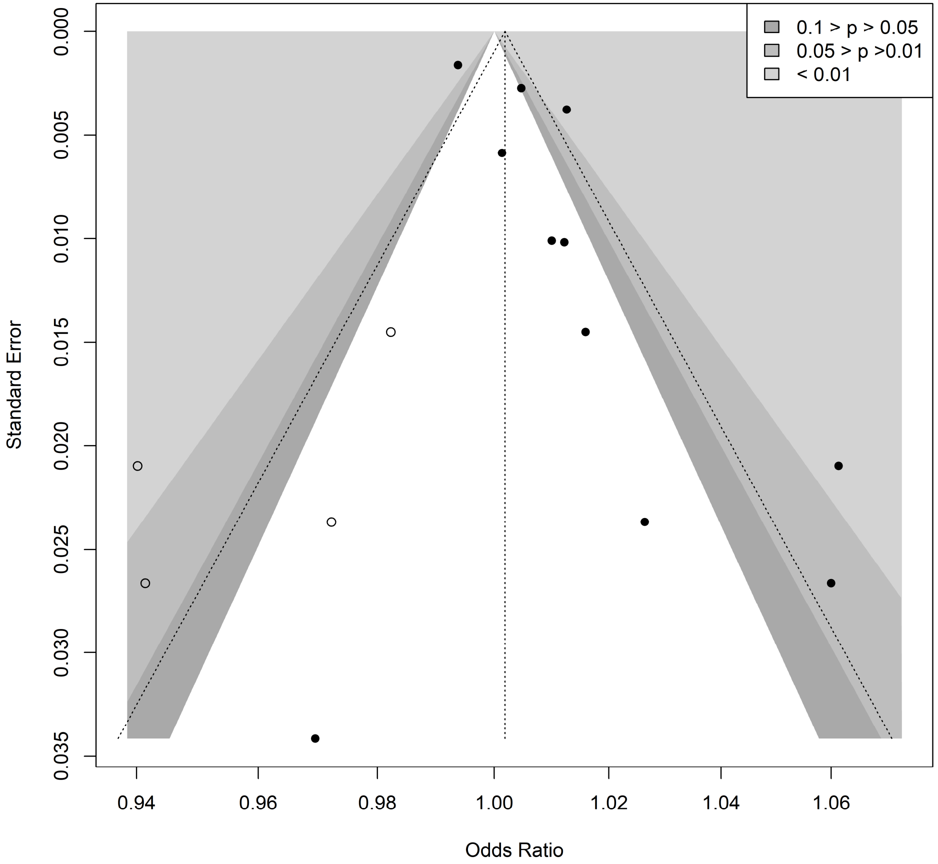

Figure S17. Funnel plot for the meta-analysis on the associations between long-term NO_2 exposure (per 1ppb increase) and risk of PD.

Abbreviations: NO_2, nitrogen dioxide; OR, odds ratio; PD, Parkinson's disease.

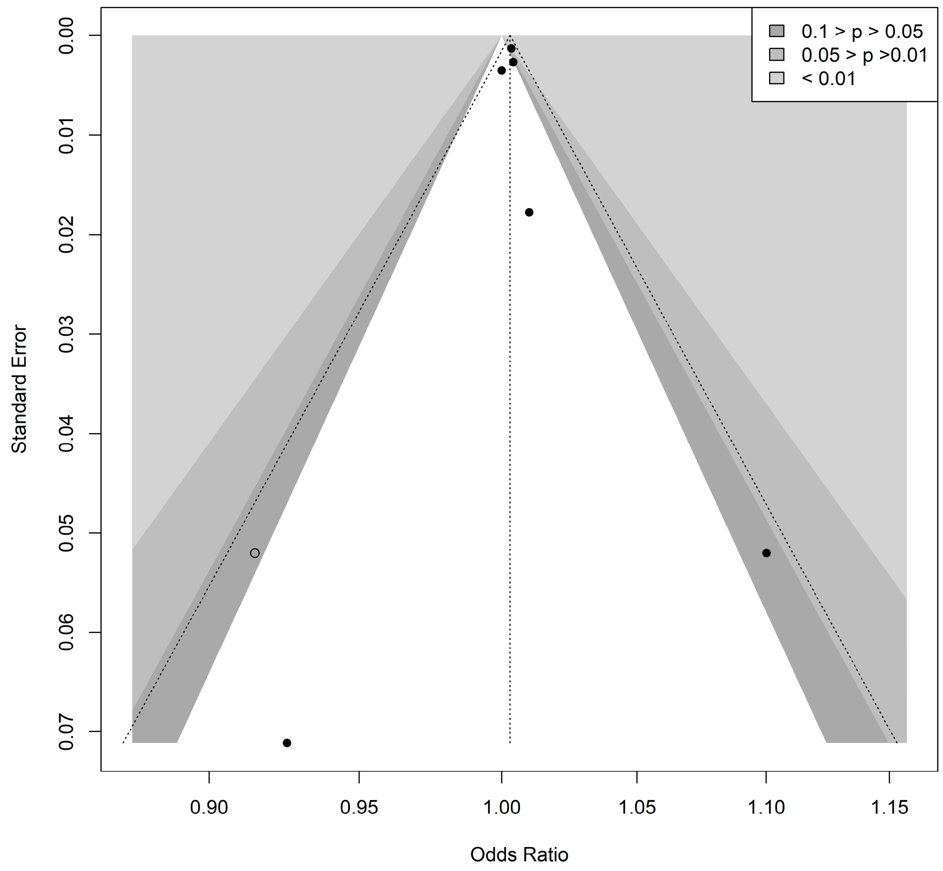

Figure S18. Funnel plot for the meta-analysis on the associations between long-term O_3 exposure (per 1ppb increase) and risk of PD.
Abbreviations: OR, odds ratio; O_3, ozone; PD, Parkinson's disease.
